# Furan Distribution as a Severity Indicator upon Organosolv Fractionation of Hardwood Sawdust through a Novel Ternary Solvent System

**DOI:** 10.1021/acssuschemeng.3c07236

**Published:** 2024-01-12

**Authors:** Petter Paulsen Thoresen, Irene Delgado Vellosillo, Heiko Lange, Ulrika Rova, Paul Christakopoulos, Leonidas Matsakas

**Affiliations:** † Biochemical Process Engineering, Division of Chemical Engineering, Department of Civil, Environmental and Natural Resources Engineering, Luleå University of Technology, SE-971 87 Luleå, Sweden; ‡ Department of Earth and Environmental Sciences, University of Milano-Bicocca, Piazza della Scienza 1, 20126 Milan, Italy; § NBFC − National Biodiversity Future Center, 90133 Palermo, Italy

**Keywords:** organosolv, lignocellulose, lignin, fractionation, ternary solvent

## Abstract

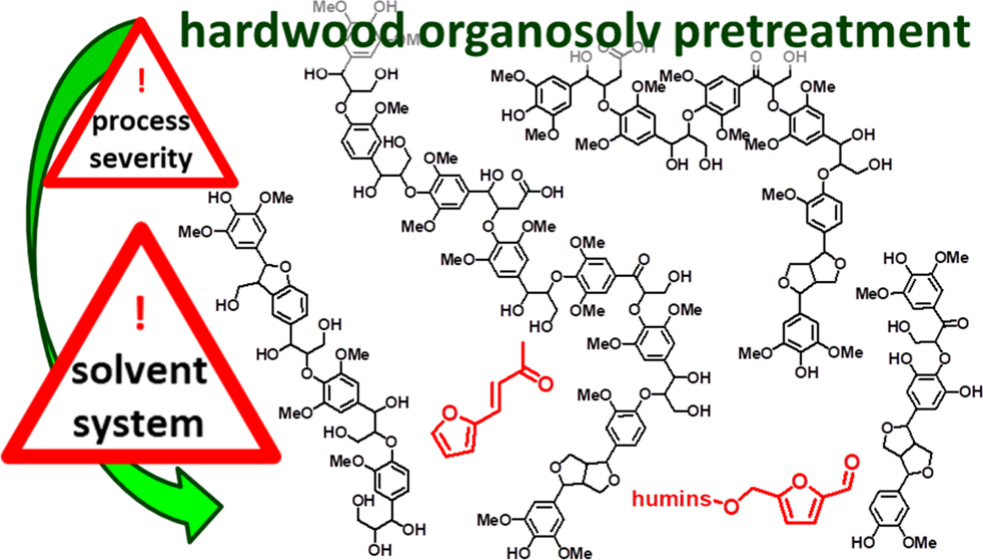

Beech sawdust was treated with a ternary solvent system based on binary aqueous ethanol with partial substitution of ethanol by acetone at four different water contents (60, 50, 40, and 30%v/v). In addition to standard, i.e., noncatalyzed treatments, the application of inorganic acid in the form of 20 mm H_2_SO_4_ was evaluated. The various solvent systems were applied at 180 °C for 60 min. The obtained biomass fractions were characterized by standard biomass compositional methods, i.e., sugar monomer and oligomer contents, dehydration product contents of the aqueous product, and lignin, cellulose, and hemicellulose contents in isolated solid fractions. More advanced analyses were performed on the lignin fractions, including quantitative ^13^C NMR analyses, ^1^H–^13^C HSQC analysis, size exclusion chromatography, and pyrolysis-GC/MS, and the aqueous product, in the form of size exclusion chromatography and determination of total phenol contents. The picture emerging from the thorough analytical investigation performed on the lignin fractions is consistent with that resulting from the characterization of the other fractions: results point toward greater deconstruction of the lignocellulosic recalcitrance upon higher organic solvent content, replacing ethanol with acetone during the extraction, and upon addition of mineral acid. A pulp with cellulose content of 94.23 wt % and 95% delignification was obtained for the treatment employing a 55/30/15 EtOH/water/acetone mixture alongside 20 mm H_2_SO_4_. Furthermore, the results indicate the formation of two types of organosolv furan families during treatment, which differ in the substitution of their C_1_ and C_5_. While the traditional lignin aryl–ether linkages present themselves as indicators for process severity for the nonacid catalyzed systems, the distribution of these furan types can be applied as a severity indicator upon employment of H_2_SO_4_, including their presence in the isolated lignin fractions.

## Abstract 2

A novel ternary organosolv solvent system was applied and revealed formation of two distinct furan families whose distribution is severity dependent.

## 1 Introduction

New research suggests that it is improbable, if not impossible, to maintain global warming underneath the ill-famous 1.5-degree threshold.^1^ This is mainly due to the ongoing consumption of fossil-based resources in gaseous, liquid, and solid forms, i.e., natural gas, oil, and coal, respectively. While the “fossil fuel” issue is well-known even to the wider public, the consumption of fossil resources in chemical industries is less known, but equally important, given the central role of the chemical industry in modern societies. A rethinking in this sector has started but is yet to find its widespread application in everyday reality. In this context, platform chemicals widely used in industry, currently obtained from fossil resources, can be readily obtained from lignocellulosic biomass.^2−4^ Several strategies are investigated in order to overcome the notorious lignocellulosic recalcitrance for proper utilization of these resources in this respect.^5−7^ Among the most promising methods are organosolv processes, which employ organic solvents with or without catalysts at elevated temperatures in order to fractionate the major lignocellulosic constituents into separate streams.^8−11^ Most processes employ binary mixtures of water alongside either ethanol or acetone.^12−16^ More recently, ternary mixtures have gained attention as a way to fractionate biomass.^17,18^ Parts of the rationale behind investigating a ternary system comprised of water/ethanol/acetone are illustrated through the enthalpies of hydration obtained for distinct lignin motifs.^19^ In the cited work, structures with primary condensed interunit motifs experienced the benefits of ethanol and acetone hydration, whereas aryl ether only benefits from acetone. Thus, to explore any potential symbiotic enhancements in terms of lignin extraction, this ternary system was chosen in the present work and is to the best of the authors’ knowledge the first work using this ternary solvent system. In the literature, various ternary solvent systems were employed for organosolv fractionation of biomasses: water/bio-oil/organic solvent,^20^ ternary systems employing deep eutectic solvents,^21^ ethyl acetate/ethanol/water,^22^ methyl isobutyl ketone (MIBK)/ethanol/water,^17^ and acetone/phenoxyethanol/water.^18^ The various systems were obviously all designed aiming at a high cellulose content in the pulp while maintaining a typical lignin characteristic. The MIBK/ethanol/water and acetone/phenoxyethanol/water systems are more interesting, with respect to this study. The MIBK/ethanol/water represents a ketone that is bulkier than the one used in this study, a fact that leads to reduced reactivity with respect to the acetone used here in combination with ethanol. The acetone/phenoxyethanol/water method uses an ethanol derivative that represents a different chemistry. We were aiming in this study at the explicit exploitation of the reactivities of both acetone and ethanol, including their hydrogen-bonding characteristics, and hence their capacities to stabilize reactive intermediates, etc.; the importance of high-performing solvent systems allowing for reduced liquid–solid ratios has already been emphasized for the economic viability of organosolv biorefineries.^23^

## 2 Materials and Methods

### 2.1 General

Chemicals and solvents applied throughout this work were purchased from VWR (Stockholm, Sweden) and Sigma-Aldrich (Burlington, MA, USA) and generally were of analytical grade. Solvents for NMR analyses were purchased as dry solvents from the same vendors. All lignins were freeze-dried or dried in a ventilated oven at 40 °C until constant weight.

### 2.2 Lignocellulosic Material

Beech sawdust LIGNOCEL HBS 150/500, obtained from Rettenmaier Sweden KB (JRS), was used. The composition of the untreated biomass was measured following a literature protocol^24^ to 22.5% w/w lignin, 37.2% w/w cellulose, and 28.5% w/w hemicellulose, with 23.50 and 5.00% w/w being sugar and acetyl groups, respectively.

### 2.3 Organosolv Pretreatment

The various treatments performed and their associated conditions are presented in Table 1. For the respective treatments, an air-heated multidigester system (Haato, Vantaa, Finland) was used.

**Table 1:** Systematic Parameter Variation of the Organosolv Treatments Performed in the Current Work

treatment code (ethanol/water/acetone)]	time [min]	temperature [°C]	ethanol/water/acetone [% v/v/v]	catalyst (sulfuric acid) [mm]
**A0-1**(40/60/0)	60	180	40/60/0	0
**A0-2**(30/60/10)	60	180	30/60/10	0
**A0-3**(20/60/20)	60	180	20/60/20	0
**A0-4**(50/50/0)	60	180	50/50/0	0
**A0-5**(40/50/10)	60	180	40/50/10	0
**A0-6**(30/50/20)	60	180	30/50/20	0
**A0-7**(60/40/0)	60	180	60/40/0	0
**A0-8**(45/40/15)	60	180	45/40/15	0
**A0-9**(30/40/30)	60	180	30/40/30	0
**A0-10**(70/30/0)	60	180	70/30/0	0
**A0-11**(55/30/15)	60	180	55/30/15	0
**A0-12**(40/30/30)	60	180	40/30/30	0
**A20-1**(40/60/0)	60	180	40/60/0	20
**A20-2**(30/60/10)	60	180	30/60/10	20
**A20-3**(20/60/20)	60	180	20/60/20	20
**A20-4**(50/50/0)	60	180	50/50/0	20
**A20-5**(40/50/10)	60	180	40/50/10	20
**A20-6**(30/50/20)	60	180	30/50/20	20
**A20-7**(60/40/0)	60	180	60/40/0	20
**A20-8**(45/40/15)	60	180	45/40/15	20
**A20-9**(30/40/30)	60	180	30/40/30	20
**A20-10**(70/30/0)	60	180	70/30/0	20
**A20-11**(55/30/15)	60	180	55/30/15	20
**A20-12**(40/30/30)	60	180	40/30/30	20

*N.B*: The treatment labels are described by their acid content (A0 or A20 for 0 or 20 mm H_2_SO_4_, respectively), and the treatment number.

After the organosolv treatment, the products in the form of liquid and residual solids were vacuum filtered, separating the product into two distinct streams: (1) The cellulose-enriched solid pulp and (2) a liquid phase comprised of dissolved lignin, sugars, and sugar derivatives. Subsequently, after isolation of the pulp, it was washed with the corresponding ternary solvent system (volume and composition) as applied during the treatment, generating a wash liquid. The respective component contents later determined through structural analysis from both liquids are in the data processing summed to give, for example, an isolated mass of lignin of both original organosolv liquor and wash liquid. The organic solvent was removed through evaporation from both the initial liquid phase and the pulp-washing solutions (processed separately) by rotary evaporation (Heidolph, Schwabach, Germany). In the processed liquid streams, precipitated lignin was recovered by centrifugation at 10,000*g* for 15 min at 4 °C (5804R; Eppendorf, Hamburg, Germany). The aqueous supernatants were stored at 4 °C in plastic bottles until further analyses. The isolated lignin was freeze-dried (Lyoquest; Telstar, Terrassa, Spain) and stored in plastic bottles at room temperature.

### 2.4 Structural Analysis of Biomass Fractions

The beech raw material, and the generated solid fractions obtained through organosolv, i.e., pulp and lignin, were analyzed for the contents of cellulose, hemicellulose, and lignin.^24^ This method is used to characterize the compositional properties of the respective fractions and will throughout be referred to as structural analysis of biomass (SAB). After SAB, the carbohydrate composition was analyzed on HPLC apparatus (PerkinElmer, Massachusetts) equipped with a Rezex RPM-monosaccharide Pb+ LC column 300 mm × 7.8 mm (Phenomenex, Värlöse, Denmark) column and a refractive index detector. The column was kept at a temperature of 85 °C with deionized water as the mobile phase at a flow rate of 0.6 mL/min.

For the obtained aqueous product, the samples were suitably diluted and analyzed directly through HPLC as described above to measure the monomeric sugar content. To evaluate the content of sugars present as oligosaccharides, the aqueous product obtained from organosolv was first hydrolyzed by treatment with H_2_SO_4_ (4% w/w) at 121 °C for 1 h, and subsequently neutralized with CaCO_3_ and then analyzed through HPLC.^25^

Recoveries are calculated in terms of components obtained relative to those originally present in the raw material. Delignification is calculated according to eq 1.
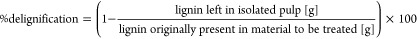
1

### 2.5 Gel Permeation Chromatography of Isolated Lignins

First, the samples were derivatized by adding 0.9 mL of glacial acetic acid and 0.1 mL of acetyl bromide to 5 mg of powdered lignin. The mixture was stirred for 2 h at room temperature in closed vials. The solution was then transferred to a round bottom flask, and the solvents were evaporated in a rotary evaporator (Heidolph, Schwabach, Germany) at 50 °C and 50 mbar. Subsequently, the sample was washed twice with 1 mL of tetrahydrofuran (THF, HPLC grade without stabilizer) followed by solvent evaporation. The sample was then dissolved in 1 mL of THF (HPLC grade without stabilizer) and filtered through 0.22 μm hydrophobic syringe filters (PTFE; Sartorius, Göttingen, Germany). Finally, the samples were analyzed by HPLC using a UV detector set at 280 nm and a StyragelHR 4E column (Waters, Milford, MA), operated at 40 °C, with THF as the mobile phase, and a flow rate of 0.6 mL/min. The calibration was done using polystyrene (Sigma-Aldrich, St. Louis, MO). The numbers were rounded up at 100 s due to the resolution of the method. The calibration curve was prepared with polystyrene standard in a molecular weight (MW) range of 0.5–90 kDa.

### 2.6 Gel Permeation Chromatography of the Aqueous Product

Samples were filtered through 0.22 μm hydrophilic filters (Sartorius, Göttingen, Germany) and analyzed using HPLC (PerkinElmer, Waltham, MA) using an RI detector and a series of two-column Ultrahydrogel 250 and 120 (Waters, Milford, MA), operated at 60 °C, with deionized water as the mobile phase, and a flow rate of 0.6 mL/min. The calibration was done by using cellobiose (MW = 342 Da, Sigma-Aldrich) and dextran (MW = 180 kDa, Sigma-Aldrich).

### 2.7 Total Phenolic Contents of the Isolated Aqueous Product

The total phenolics content in the isolated aqueous products was determined as described previously^26,27^ with the exception of applying ethanol instead of methanol and expressed as the mass of gallic acid equivalents (GAEs).

### 2.8 Quantitative ^13^C NMR Analysis

Accurately weighted lignin samples of ∼80 mg were dissolved in 500 μL of DMSO-*d*_6_, and 50 μL of Cr(III) acetylacetonate in DMSO-*d*_6_ (∼1.5 mg/mL) were added as a spin-relaxation agent, and 50 μL of trioxane (92.92 ppm) in DMSO-*d*_6_ (∼15 mg/mL) was used as an internal standard. Spectra were recorded at room temperature on a Bruker 600 MHz Avance III spectrometer (Bruker Biospin) controlled with Topspin 3.6.4 and equipped with a 5 mm BBO broadband (1*H*/19*F*/2D) z-gradient cryo-probe. An inverse-gated proton decoupling pulse sequence was applied with a 90° pulse width, a relaxation delay of 1.7 s, and an acquisition time of 1.2 s. A total of 40 000–48 000 scans were acquired for each spectrum. NMR data were processed using MestreNova Version 9.0.1 (Mestrelab Research S.L.).

### 2.9 ^1^H–^13^C Heteronuclear Single Quantum Coherence (HSQC) Analysis

^1^H–^13^C heteronuclear single quantum coherence (HSQC) analysis was performed on selected lignins. The NMR samples were the same as those used for the quantitative ^13^C NMR analyses of the selected lignins. The spectra were obtained at 30 °C on a Bruker 600 MHz Avance III spectrometer (Bruker Biospin) controlled with Topspin 3.6.4 and equipped with a 5 mm BBO broadband (1*H*/19*F*/2D) z-gradient cryo-probe. The Bruker hsqcetgpsisp2.2 pulse program in DQD acquisition mode was used, with NS = 64; TD = 2048 (F2) and 512 (F1); SQ = 12.9869 ppm (F2) and 164.9996 ppm (F1); O2 (F2) = 2601.36 Hz and O1 (F1) = 7799.05 Hz; D1 = 2 s; CNST2 1J(C–H) = 145; and acquisition time F2 channel = 197.0176 ms and F1 channel = 15.4164 ms. NMR data were processed using MestreNova.

### 2.10 Pyrolysis-Gas Chromatography–Mass Spectroscopy (pyr-GC/MS)

Pyr-GC/MS was carried out on a Frontier Lab PY-3030S pyrolyzer, using 600 °C as the pyrolyzing temperature. The pyrolyzer was coupled to a PerkinElmer Clarus GC/MS 690/SQ8T-equipped with a Restek RTX-1701 column (60 m × 0.25 mm, i.d. 0.25 μm film thickness) and a quadrupole mass spectrometer detector (EI at 70 eV, ion source at 240 °C). A split ratio of 1:10 and an injection temperature of 280 °C were used. The temperature in the chromatograph oven was initially held at 40 °C for 1 min, then ramped at 8 °C/min to 280 °C, and held there for 45.00 min. Helium at a flow rate of 1.0 mL/min was used as the carrier gas. Mass spectra in the molecular mass range *m*/*z* = 50–400 were obtained.

### 2.11 Determination of Degradation and Dehydration Compounds in the Aqueous Product

Contents of dehydration products and organic acids were determined by HPLC (PerkinElmer, Waltham, MA) with an ultraviolet (UV) detector set at 205 (acetic acid), 227 (formic acid), or 280 nm (furfural, 5-hydroxymethyl furfural (5-HMF), levulinic acid) connected with an Aminex HPX-87H column (Bio-Rad, Hercules, CA) operated at 65 °C, with 0.005 M H_2_SO_4_ as the mobile phase, and a flow rate of 0.6 mL/min.

## 3 Results and Discussion

In order to present a coherent and progressive picture of the fractionation process, results are discussed in sections: first, the obtained pulps are characterized; subsequently, the two remaining fractions, i.e., isolated lignin and aqueous products, are described in light of the previously depicted data. The rationale behind this is that describing the pulp furnishes a broad overview and introduction considering the extent of the fractionation process, whereas characterizing the two fractions provides details of the “severity” of the process when, for example, looking into both native and process-induced chemistries.

### 3.1 Pulp Composition

The obtained pulps from the various organosolv treatment conditions (Table 1) are described in detail in Table 2.

**Table 2:** Obtained Pulps from Treatments Performed without Mineral Acid and Their Characteristics

treatment code	pulp recovery [%m/m]	%Lignin in pulp (%recovery) [%m/m]	delignification [m/m]	%cellulose in pulp (%recovery) [%m/m]	%hemicellulose in pulp (%recovery) [%m/m]
**A0-1**	61.41	21.72 (59.26)	0.41	57.67 (95.22)	7.17 (30.51)
**A0-2**	57.39	20.90 (53.29)	0.47	54.36 (83.90)	8.33 (35.46)
**A0-3**	62.49	18.12 (50.30)	0.50	60.20 (101.15)	7.15 (30.43)
**A0-4**	60.94	14.52 (39.29)	0.61	56.69 (92.89)	11.29 (48.03)
**A0-5**	60.54	13.15 (35.37)	0.65	56.64 (92.21)	11.12 (47.31)
**A0-6**	62.92	15.17 (42.40)	0.57	58.23 (98.52)	13.82 (58.80)
**A0-7**	74.35	13.56 (44.80)	0.55	51.05 (102.07)	15.88 (67.59)
**A0-8**	72.20	13.28 (42.59)	0.57	49.15 (95.43)	15.71 (66.86)
**A0-9**	71.70	13.19 (42.03)	0.59	51.56 (99.41)	15.42 (65.64)
**A0-1**	82.69	15.60 (57.29)	0.43	46.73 (103.91)	20.97 (89.23)
**A0-11**	88.76	15.32 (60.40)	0.40	47.29 (110.02)	22.52 (95.82)
**A0-12**	84.19	15.62 (58.41)	0.52	47.34 (107.16)	20.26 (86.22)
**A20-1**	40.56	21.30 (38.38)	0.62	74.70 (81.46)	0.00 (0.00)
**A20-2**	37.34	21.81 (36.18)	0.64	75.55 (75.86)	0.00 (0.00)
**A20-3**	37.47	22.46 (37.38)	0.63	75.21 (75.77)	0.00 (0.00)
**A20-4**	37.48	11.64 (19.38)	0.81	82.66 (83.31)	0.00 (0.00)
**A20-5**	32.94	10.32 (15.10)	0.85	84.22 (74.61)	0.00 (0.00)
**A20-6**	31.98	8.92 (12.68)	0.87	84.45 (72.62)	0.00 (0.00)
**A20-7**	31.82	5.26 (7.44)	0.93	85.53 (73.17)	0.00 (0.00)
**A20-8**	28.14	4.61 (5.77)	0.94	87.29 (66.04)	0.00 (0.00)
**A20-9**	24.25	5.46 (5.88)	0.94	91.65 (59.76)	0.00 (0.00)
**A20-10**	27.56	5.61 (6.86)	0.93	92.00 (68.17)	0.00 (0.00)
**A20-11**	24.66	5.05 (5.53)	0.95	94.24 (62.49)	0.00 (0.00)
**A20-12**	21.18	5.56 (5.23)	0.95	91.17 (51.92)	0.00 (0.00)

*N.B*: The treatment labels are described by their acid content (A0 or A20 for 0 or 20 mm H_2_SO_4_, respectively), and the treatment number.

In terms of fractionation performance, the two highest water contents (60 and 50%v/v) present the lowest pulp recoveries of hemicellulose and lignin. At lower water contents, the nonacid catalyzed systems eventually experience a stagnant delignification potentially due to improper fractionation of the hemicellulose moieties.^28,29^ Thus, in the absence of acid, higher water contents appear to favor the extraction of hemicellulosic sugars, whereas the highest water content is unfavorable for lignin extraction. Moving from 60 to 50%v/v water appears to overcome this to a certain extent, while further reduction of the water content limited the extraction of C5 sugar components, which in turn eventually limits lignin extraction.

In a rough comparison, neglecting the potential effects sulfuric acid can have on properties other than depolymerization and formation of soluble polysaccharide derivatives, this is further indicated by the pulp characteristics obtained when using 20 mm H_2_SO_4_ (**A20** series) where the only limitation on delignification is observed at the highest water contents likely originating from solubility effects. Another result worth mentioning upon employment of acid is the gradual decrease in cellulose recovery in the pulp (Table 2; cellulose mass balances given in Tables S2.2.1 and S.2.2.2) upon stepwise addition of acetone, potentially due to the formation of glucoseptanosides^30^ or similar byproducts previously reported to form when alcohols and acetone are introduced alongside sugars in acidic environments.^31^

### 3.2 Lignin Characterization

Prior to structural elucidations, the residual sugar contents in isolated lignins were determined through acid hydrolysis of the obtained lignin fractions. The sugar residues were further distinguished based on their origin from either cellulose or hemicellulose. As can be seen in Tables S1.1 and S1.2, the lignin purity (g Klason lignin/g raw lignin) is consistently high in terms of Klason lignin, with contents being generally in the range of 0.85–0.92, with few exceptions (**A0-8** with Klason lignin content of 0.83 g/g, and the wash lignin of **A0-3** with a Klason lignin content of 0.76 g/g). Correspondingly, the residual sugar contents are generally low. It is higher in the lignins obtained through nonacid catalyzed organosolv processes. The lignin mass balances are presented in Tables S2.1.1 and S2.1.2. Here, it should be noted that for selected treatments, the mass balances reach well beyond 100%, which hints toward the formation of pseudolignins^32,33^ or humin^34,35^ structures. However, as will be further discussed in the following sections, this is not contradictory to the results as a whole.

Gel permeation chromatography was performed to unravel the molecular size distributions for the isolated lignins (Table 3). For the lignins obtained from the nonacid catalyzed solvent systems, i.e., the **A0** series, the Mn generally decreased upon partial replacement of ethanol with acetone, except for the lowest water content for which a small increase in Mn is seen. For the acid-catalyzed system, i.e., the **A20** series, results for 60% v/v (**A20-1,2,3**) and 30% v/v water (**A20-10,11,12**) are similar, with the addition of acetone resulting in size reduction and increase, respectively. At the two intermediate water levels, the changes are less obvious. The introduction of acetone increases the dispersity index and/or molecular weights rather than decreasing them. The reason for this is likely complex; yet, an explanation could be closely related to cross-linking events incorporating carbonyl functionalities, as discussed in the next section (*vide infra*). Molecular weights obtained from the organosolv processes described herein are, however, generally in good agreement with those obtained for lignin from beech obtained through organosolv, as reported before.^36^

**Table 3:** Molecular Weights Obtained for Lignins from Acid-Free/Acid-Catalyzed Organosolv Treatments

treatment	Mn [Da]	Mw [Da]	DI	treatment	Mn [Da]	Mw [Da]	DI
**A0-1**	1000	2300	2.30	**A20-1**	1500	4200	2.80
**A0-2**	800	1900	2.38	**A20-2**	1300	3700	2.85
**A0-3**	800	1700	2.13	**A20-3**	1200	3300	2.75
**A0-4**	2100	11400	5.43	**A20-4**	1600	7300	4.56
**A0-5**	1800	7500	4.17	**A20-5**	1500	7200	4.80
**A0-6**	1800	9400	5.22	**A20-6**	1600	7500	4.69
**A0-7**	1900	8000	4.21	**A20-7**	1400	6500	4.64
**A0-8**	1700	6900	4.06	**A20-8**	1400	7200	5.14
**A0-9**	1600	6500	4.06	**A20-9**	1700	7100	4.18
**A0-10**	1100	3400	3.09	**A20-10**	1200	4200	3.50
**A0-11**	1200	3500	2.92	**A20-11**	1300	4600	3.54
**A0-12**	1200	4000	3.33	**A20-12**	1200	4600	3.83

In order to obtain an in-depth structural view of the isolated lignin fractions, both pyr-GC/MS and ^13^C NMR and HSQC analyses were performed on a representative selection of isolated lignins. For both the acid and nonacid catalyzed treatments, two lignins were chosen for each water concentration, namely, the one obtained from the respective binary water/ethanol system, and the one isolated upon 20%v/v acetone addition. For pyr-GC/MS analysis, only signals above a detection threshold of 0.50 were included (Tables S4.1 and S4.2), with exceptions made for the furan-based signals due to their (potentially) important role in the structural modifications of isolated lignin. Pyr-GC/MS data suggest that for the nonacid catalyzed system, i.e., the **A0** series, the strongest signal consistently originates from 4-hydroxy-3,5-dimethoxy-benzaldehyde. For the three treatments **A20-1**, **A20-3**, and **A20-4**, the largest signal originates from 4-ethyl-2-methoxyphenol. Meanwhile, upon a further increase of organic solvent content in the **A20** series, larger contributions are obtained from sugar dehydration products. This is seen, for example, for treatments **A20-6**, **A20-9**, **A20-10**, and **A20-12**, where the largest individual signal originating from 2-furanyl-3-buten-2-one or 5-hydroxymethyl furfural (5-HMF), respectively. 2-Furanyl-3-buten-2-one is also observed for the nonacid catalyzed A0 systems that employed acetone, with this structure being a product of the reaction between acetone and furfural aldehyde.^37^ A second signal, which is interesting in this context is the one automatically assigned to 1,2,3,4-tetramethoxy-5-(2-propenyl)-benzene, but more realistically representing lignin-derived 2,6-dimethoxy-4-(1-ethoxyprop-2-en-1-yl)-phenol, this structure is present only in the nonacid catalyzed **A0** systems. Also interesting is that these signals decrease or disappear upon partial replacement of ethanol with acetone, whereas the signal for 2-furanyl-3-buten-2-one augments. This could suggest that furfural aldehyde can react either with acetone or with phenolic ring carbons.

The G/S/H ratios presented in Tables S4.1 and S4.2 are based on the pyr-GC/MS results. In general, the nonacid catalyzed **A0** systems yield values, which are to be expected from hardwood.^38^ At lower water contents, especially the G/S contents appear to approach unity, which is also observed upon employment of the mineral acid catalyst in the **A20** series. Considering also the other data presented in Table S4.1 and S4.2 and the structures obtained from the nonacid catalyzed **A0** organosolv lignins, there are no dominating signals following the changes in solvent systems as seen during acid employment (Table S4.2); instead, there is a consistent increase and decrease throughout for the structures associated with G or S units, respectively.

The trends for the H/G/S ratios as delineated by the pyr-GC/MS data are less obvious when observing the HSQC data (Tables 4 & 5), with shifts used for assignment presented in Table S5.1. Structures discussed in the tables are shown in Figure 1, in which the case of furan motifs are indicated, both the structures seen in the HSQC spectra as well as the derivatives thereof detected in pyr-GC/MS. Assignments within the HSQC spectra are shown as an example in Figure 2, displaying spectra of samples **A0-1** and **A20-1**. The HSQC spectra and the quantitative ^13^C NMR spectra used for the quantification of the HSQC spectra are shown in the Supporting Information (Figures S1–S16).

**Figure 1:**
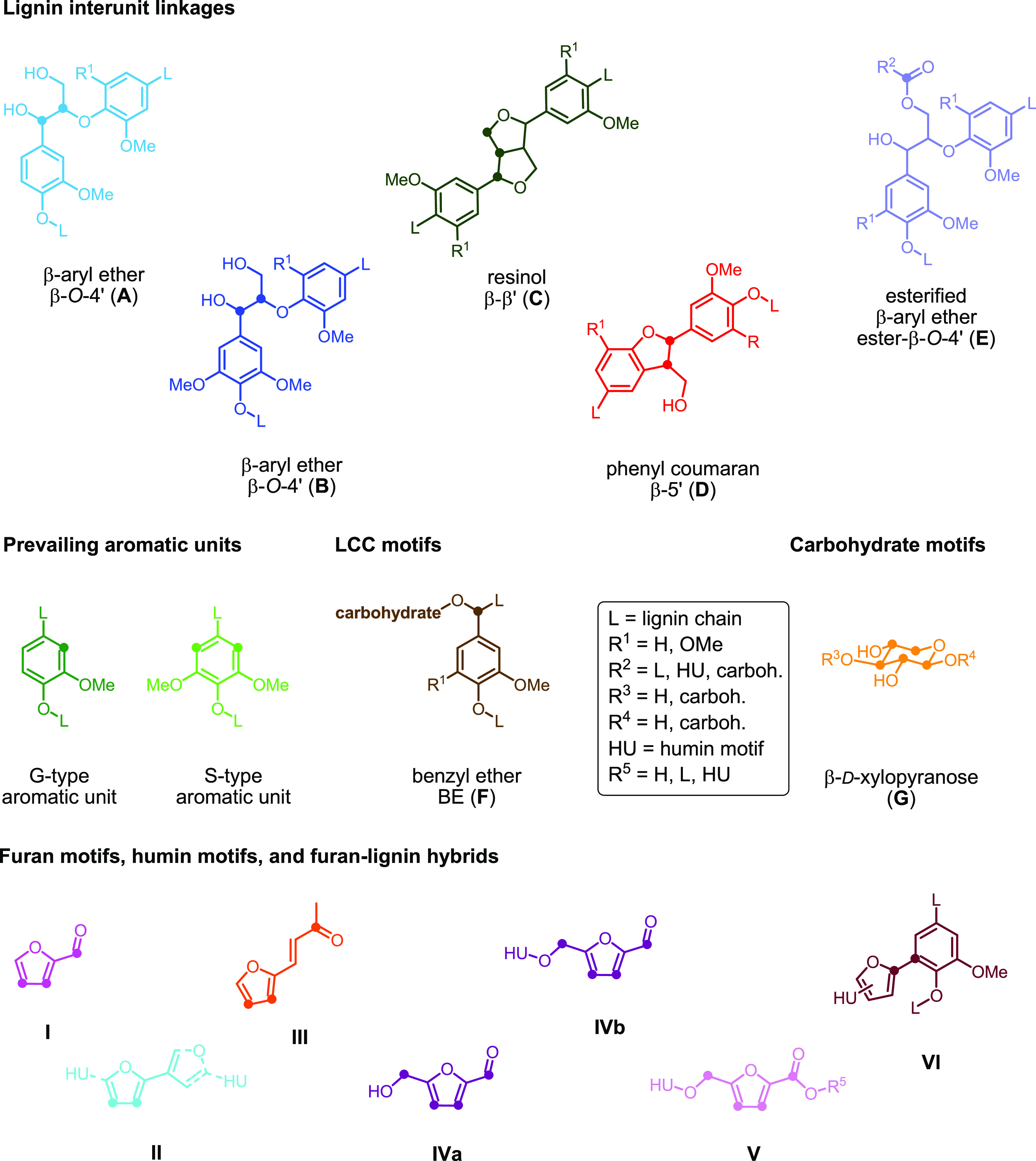
Important structural motifs of lignins unambiguously identified in the HSQC analysis and ^13^C NMR spectra of the lignins discussed. Color coding matches that used in Figure 2. Positions marked with a dot have been used for assignments and semiquantitative evaluations. Dashed lines indicate parts of the structure that got lost during the pyrolysis, leading to the solid drawn fragment detectable in pyr-GC/MS.

**Figure 2:**
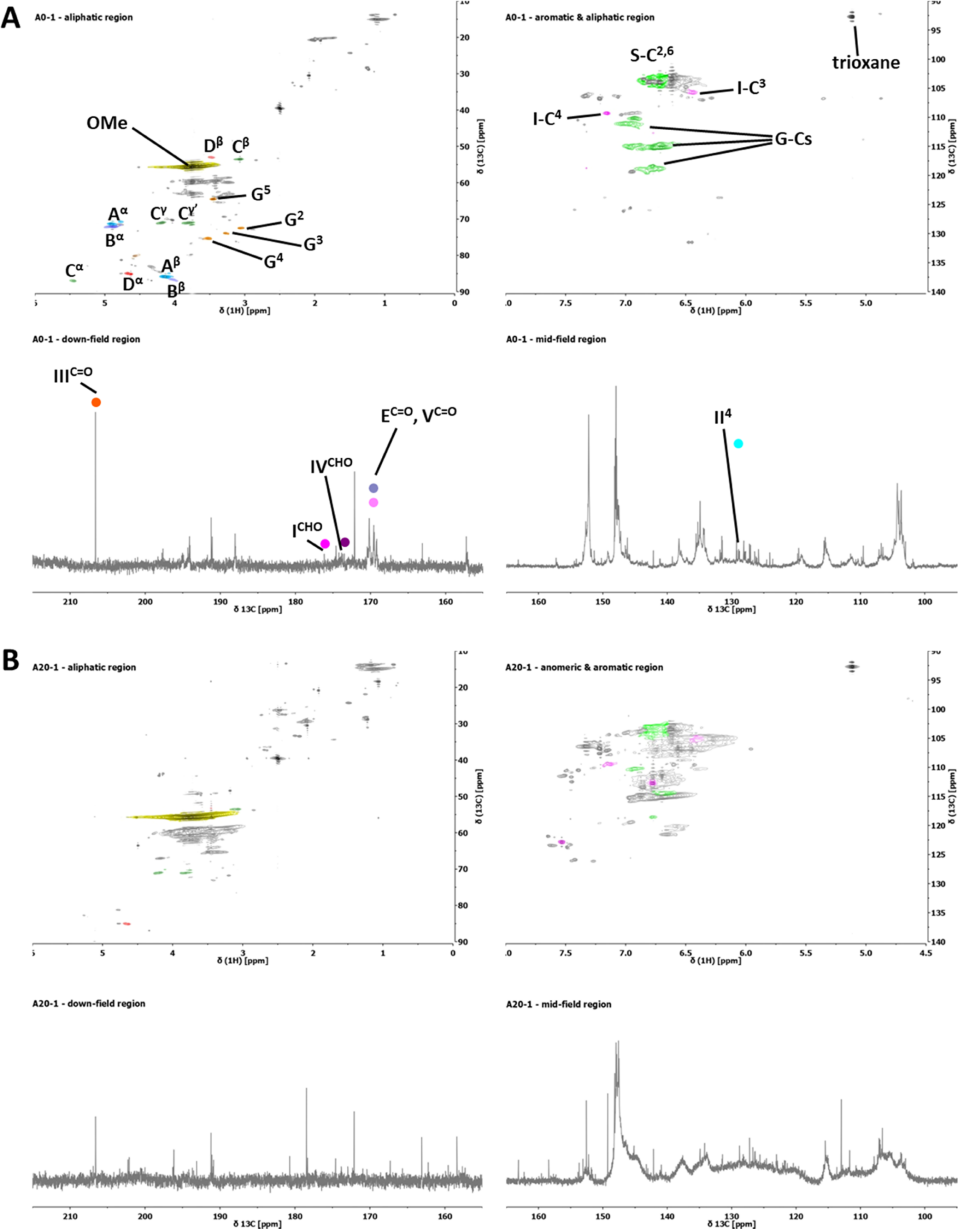
HSQC spectra of the isolated lignins, split into aliphatic and anomeric/aromatic regions. Key structural motifs were color-coded following colors used in Figure 1: (A) **S1**; (B) **S2**; and (C) **S3**.

**Table 4:** Functional Groups Content for Selected Lignins Isolated Using Acid-Free Organosolv Treatments (**A0** series). Data Have Been Derived by Quantifying HSQC Spectra on the Basis of Quantitative ^13^C NMR Measurements^35,40^

motif	**A0-1** [mmol/g]	**A0-3** [mmol/g]	**A0-4** [mmol/g]	**A0-6** [mmol/g]	**A0-7** [mmol/g]	**A0-9** [mmol/g]	**A0-10** [mmol/g]	**A0-12** [mmol/g]
G-ring (HSQC)	1.11	1.25	1.20	1.24	0.75	0.74	1.10	1.07
S ring (HSQC)	2.78	3.02	3.27	3.51	2.26	1.97	2.22	2.69
-OMe (^13^C)	11.95	11.77	14.23	11.10	10.67	10.45	11.33	11.29
Quat. Phe C-OMe (^13^C) (V)	12.50	12.88	14.25	10.71	10.65	10.37	12.20	11.80
β-*O*-4′ (G) (HSQC) (A)	0.39	0.36	0.50	0.45	0.39	0.37	0.36	0.32
β-*O*-4′ (S) (HSQC) (B)	0.94	0.84	1.04	0.80	0.79	0.74	0.94	0.93
β–β′ (HSQC) (C)	0.38	0.51	0.75	0.59	0.55	0.49	0.56	0.56
β-5′ (HSQC) (D)	0.17	0.18	0.24	0.13	0.18	0.15	0.19	0.17
β-d-Xylanopyr. (HSQC) (G)	0.18	0.15	0.24	0.14	0.18	0.15	0.12	0.11
BE (HSQC) (F)	0.08	0.05	0.09	0.06	0.08	0.06	0.06	0.05
furan C3 type 1 (HSQC)^a^ (I, II, III)	0.51	0.68	0.92	0.87	0.55	0.51	0.53	0.66
furan C4 type 1 (HSQC) (I, II, III)	0.66	0.72	1.06	1.03	0.66	0.52	0.59	0.68
furan C3 type 2 (HSQC) (IVa, IVb, V)	0.00	0.00	0.00	0.00	0.00	0.00	0.00	0.00
furan C4 type 2 (HSQC) (IVa, IVb, V)	0.00	0.00	0.00	0.00	0.00	0.00	0.00	0.00
ketone (^13^C) (III)	0.06	0.04	0.00	0.00	0.00	0.01	0.00	0.02
ester (^13^C) (E)	0.53	0.61	0.81	0.65	0.59	0.55	0.51	0.50
furan aldehyde (HSQC) (I, IVa, IVb)	0.00	0.00	0.00	0.00	0.00	0.00	0.00	0.00
S:G (pyr-GC/MS)	71:29	71:29	73:27	74:26	75:25	73:27	67:33	71:29

a Potential overlap. Furan C_3,4_ and overlap with substituted G. Not included in the Phe/fur ratio (HSQC).

**Table 5:** Functional Groups Content for Selected Lignins Isolated Using Acid-Free Organosolv Treatments (A20 Series). Data Have Been Derived by Quantifying HSQC Spectra on the Basis of Quantitative ^13^C NMR Measurements^35,40^

motif	**A20-1** [mmol/g]	**A20-3** [mmol/g]	**A20-4** [mmol/g]	**A20-6** [mmol/g]	**A20-7** [mmol/g]	**A20-9** [mmol/g]	**A20-10** [mmol/g]	**A20-12** [mmol/g]
G-ring (HSQC)	0.12	0.10	0.39	0.23	0.11	0.09	0.06	0.04
S ring (HSQC)	0.58	0.62	0.73	0.67	0.27	0.22	0.54	0.57
-OMe (^13^C)	8.72	8.34	8.29	9.22	7.51	7.16	8.07	7.05
Quat. Phe C-OMe (^13^C) (V)	8.72	8.12	9.24	10.12	8.40	7.76	9.27	9.42
β-*O*-4′ (G) (HSQC) (A)	0.00	0.00	0.00	0.00	0.00	0.00	0.00	0.00
β-*O*-4′ (S) (HSQC) (B)	0.00	0.00	0.00	0.00	0.00	0.00	0.00	0.00
β-β′ (HSQC) (C)	0.07	0.05	0.11	0.09	0.08	0.07	0.07	0.04
β-5′ (HSQC) (D)	0.00	0.00	0.00	0.00	0.00	0.00	0.00	0.00
β-d-Xylanopyr. (HSQC) (G)	0.00	0.00	0.00	0.00	0.00	0.00	0.00	0.00
BE (HSQC) (F)	0.00	0.00	0.00	0.00	0.00	0.00	0.00	0.00
furan C3 type 1 (HSQC)^a^ (I, II, III)	1.08	0.72	2.78	1.13	0.54	0.49	0.65	0.64
furan C4 type 1 (HSQC) (I, II, III)	0.32	0.26	0.70	0.55	0.21	0.24	0.29	0.37
furan C3 type 2 (HSQC) (IVa, IVb, V)	0.44	0.38	1.08	0.95	0.39	0.48	0.65	0.76
furan C4 type 2 (HSQC) (IVa, IVb, V)	0.12	0.13	0.42	0.51	0.20	0.27	0.40	0.44
ketone (^13^C) (III)	0.03	0.31	0.05	0.12	0.21	0.40	0.16	0.47
ester (^13^C) (E)	0.00	0.00	0.00	0.00	0.00	0.01	0.01	0.02
furan aldehyde (HSQC) (I, IVa, IVb)	0.07	0.14	0.14	0.32	0.20	0.34	0.47	0.47
S:G (pyr-GC/MS)	83:17	86:14	65:35	74:26	71:29	71:29	90:10	93:7

a Potential overlap. Furan C_3,4_ and overlap with substituted G. Not included in the Phe/fur ratio (HSQC).

To correlate the quantitative ^13^C NMR data with the HSQC for nonacid catalyzed treatments (**A0** samples), the signal attributed G_2_ was used for the aromatics, while β-*O*-4′ (C_α_) was used for the aliphatic. For the acid-catalyzed treatments (**A20** samples), the S_2,6_ signal was used for the aromatics, while the β-β signal (C_α_) was used for the aliphatic signals. The overall content of methoxy groups was estimated through ^13^C and found to generally match the content of quaternary **C**-OMe species.^39^

In contrast to the pyr-GC/MS data, quantified HSQC data suggest H/G/S ratios typical of a hardwood. With this being understood based on the fact that the pyrolysis generates derivatives not necessarily directly stemming from units originally present in the starting material, the comparison across analysis techniques could hence give hints as to which structures can actually give rise to detectable fragments upon pyrolysis. Considering some of the structural motifs delineated on the basis of the NMR analysis, the content of syringyl units augments alongside the frequency of β-β′ interunit structures, which in turn had been previously proposed to be predominantly formed within S-lignins.^41^

**Figure 3:**
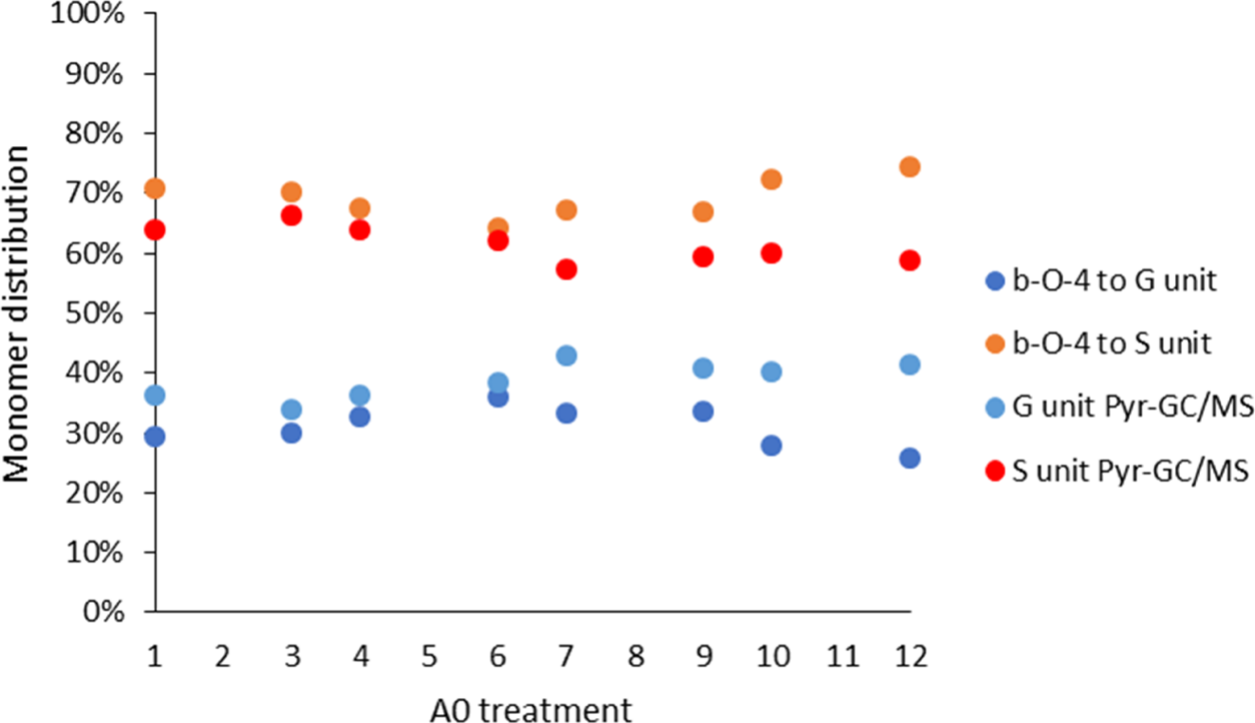
Development of the lignin monomer ratios across the **A0** series of lignins as delineated from quantified HSQC using the β-*O*-4′ motif and pyr-GC/MS.

If instead considering the β-*O*-4′ structures as those most readily available to form fragments when exposed to temperatures used during the pyrolysis process, the S and G unit ratios follow a relatively close interrelation when comparing the values obtained on specific β-*O*-4′ content and monomers released upon pyr-GC/MS (Figure 3). Here, the closest match is found at the two lowest water contents (**A0-1** and **A0-6** treatments in Figure 3).

As seen in the form of the GPC data generated for the lignins, at lower water contents, the lignins are dominated by lower molecular weight structures. Considering that benzyl-ether bond cleavage in the case of syringyl units is considered slower than that of guaiacyl-linked benzyl-ethers,^42^ this finding suggests that the difference observed at lower water contents could originate from G-units already depolymerized, and thus more easily forming detectable fragments upon pyrolysis. This would, in turn, indicate an overestimation of their contents in pyr-GC/MS analyses, and explain the increasing deviation observed.

Considering the overall content of G or S units, their concentration lies at around 1/3 of the total phenolic content when no acid is applied during the extraction. Upon the application of acid, this content decreases significantly.

To further investigate the reason behind this, the data obtained from pyr-GC/MS indicating sugar derivatives (Table 6, compare also Tables S4.1 and S4.2) were used alongside furan-stemming HSQC signals to estimate the ratio between two types of furan rings.

**Table 6:** Sugar-Derived Motifs Identified in the pyr-GC/MS Analysis of **A20** Lignins^a^

	content in samples [%A]^b^							
identified compound	**A20-1**	**A20-3**	**A20-4**	**A20-6**	**A20-7**	**A20-9**	**A20-10**	**A20-12**
furfural	2.29	2.98	2.91	4.97	4.43	4.91	5.36	3.71
5-HMF	0.79	1.09	1.66	2.01	4.35	4.08	7.30	4.31
furan, 2-(2-propenyl)-	ND^c^	1.26	ND	1.10	ND	1.62	ND	1.27
2-furancarboxaldehyde, 5-methyl-	ND	0.76	0.57	0.79	1.16	1.48	1.68	0.94
3-buten-2-one, 4-(2-furanyl)-	ND	3.99	ND	4.09	ND	5.46	ND	3.80
1-heptanone, 1-(2-furanyl)-	ND	ND	ND	ND	ND	ND	0.27	ND
4,5,6-trimethoxy-7-methyl-3*H*-2-benzofuran-1-one;	ND	ND	ND	ND	ND	ND	ND	ND
benzene, 1,2,3,4-tetramethoxy-5-(2-propenyl)-								
α-d-glucopyranose, 1,6-anhydro	0.63	ND	ND	ND	0.77	ND	0.74	0.98

a *N.B.*: Names as Listed in the NIST Database Have Been Used.

b %area: Corresponding area for a compound relative to the sum of all the signal areas in the chromatogram.

c ND: indicates that a compound is below the detection limit of %area = 0.5.

**Figure 4:**
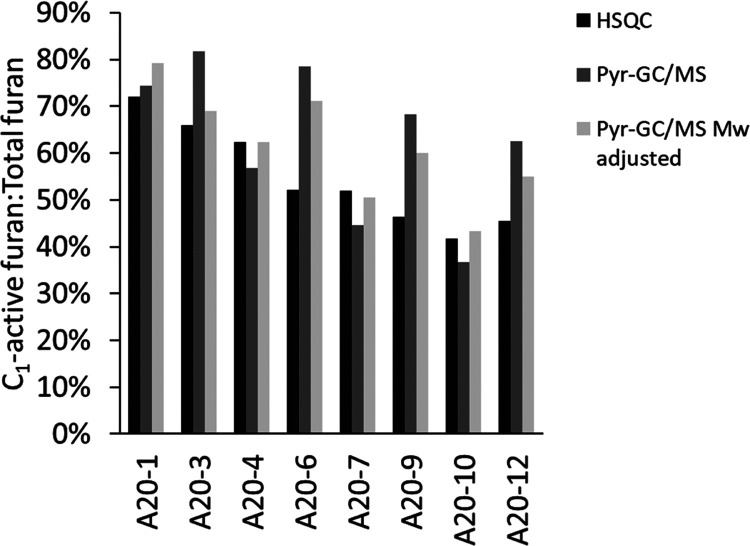
Percentage of active C_1_ (with free C_5_) vs total furan content. The ratio obtained from HSQC is based on the furan ring ratio, whereas the initial ratio from Pyr-GC/MS is on a mass basis. In addition, a third ratio from the pyr-GC/MS data is included where the molecular mass of the fragments is used to normalize the masses. In short, on the sorting of pyr-GC/MS compounds: 2-(2-propenyl)-furan (active), furfural (active), 4-(2-furanyl)-3-buten-2-one (active), 5-HMF, and (2-furancarboxaldehyde, 5-methyl). For the HSQC integration, the carbons are measured as C_4_ for the “active” furan by the signal at 7.28/106.38 ppm, whereas the signal at 7.53/122.86 ppm is integrated as C_4_ for the second type of furan.

Whereas the HSQC shifts for the 5-HMF stemming aldehyde and hydroxymethyl moieties match with signals already assigned elsewhere,^43^ the signals shifted upfield by 2–4 ppm (carbon domain) of S2,6 at δ_H_ = 7.2 ppm, are still unassigned. However, as already elucidated,^44^ these can also have furan origin, depending on the chemistry the C_2_ and C_5_ carbons undergo; the mentioned shift often occurs due to furan C_1_ carbonyl functionality while C_5_ remains “free”. Hence, two distinct types of furans seem to be present in the isolated lignins. Next, the regions occurring at δ_C_ = 106 ppm were integrated in the HSQC and quantified on the basis of the quantitative ^13^C NMR analyses of the very same sample; it is suggested that these originate from furans with at least a free aldehyde or another carbonyl/conjugate functionality (Type 1) while the furan moiety at higher carbon shifts are different, potentially because the aldehyde or ester functionality is lost (Type 2) and/or due to aliphatic substitution at C_5_, as seen for 5-HMF, for example. A similar approach can be taken for the analysis of the pyr-GC/MS data with respect to furan moieties (Table 5), considering the pyrolysis products of furfural, i.e., 2-(2-propenyl)-furan and, 4-(2-furanyl)-3-buten-2-one, as indicators of a functional C_1_, or a C_1_ side-chain with an aldehyde or conjugation, and assuming a comparable ionization potential for these substances. Further, combining the signals from these products and generating a ratio against the total amount of furan moieties gives a means to correlate the data obtained from NMR to those from pyr-GC/MS. The ratios are presented in Figure 4.

Overall, the rather good correlation seems to support the idea that these signals are indeed of furan origin. However, upon the addition of acetone, volatile C_1_ active furan moieties seem to dominate in pyr-GC/MS, which could be due to the reactivities of their predecessors with less volatile and polymeric structures in the absence of acetone. Notably, both within the acid-catalyzed system and the nonacid catalyzed treatments, system reactivity appears to increase with reduced water content as the presence of functional furans decreases.

While it is important to describe the signals originating from furan moieties due to their obvious presence in the HSQC, more “traditional” lignin and sugar motifs are also present. For example, the carbon–proton shifts originating from xylose are present in the treatments performed in the absence of acid at contents of around 0.200 mmol/g, which is equivalent to 3.00 wt % which matches the mass balance data presented in Table S2.3.1. Common to all lignins isolated in the absence of mineral acid but upon the addition of acetone is the decrease in the β-*O*-4′ content, whereas generally, with the exception of **A0-7** and **A0-9**, the content of lignin monomers increases or remains constant, suggesting a preference for lignin depolymerization upon acetone addition. Considering the traditional motifs, upon the addition of mineral acid, only the resinol signals are persevered, indicating severe depolymerization and potential modification. For the latter, support can be found in the number of methoxy groups compared to the quaternary phenolic ring carbons linked to the respective OMe-group, which throughout the **A0** treatments match reasonably. Meanwhile, upon acid employment and higher organic solvent content, the actual OMe-group content is lower than the quaternary ring carbon content, suggesting other substituents than methoxyl groups. For the monomeric content of the isolated lignins in the **A20** series, there is a decrease in the content of guaiacyl groups upon application of acetone, implying their modification, potentially through increased cross-reactions upon increased reactivity as seen in the **A0** series. Interestingly, upon increasing the content of the organic solvent, a decrease in furan motifs with a free C_5_ and with C_1_ functionality in the form of carbonyl or conjugation is observed, suggesting that a greater content reacts further as the water content decreases. For every addition of acetone, there is an increase in branched furans, probably due to the cross-reaction of acetone and the furan aldehyde.

**Figure 5:**
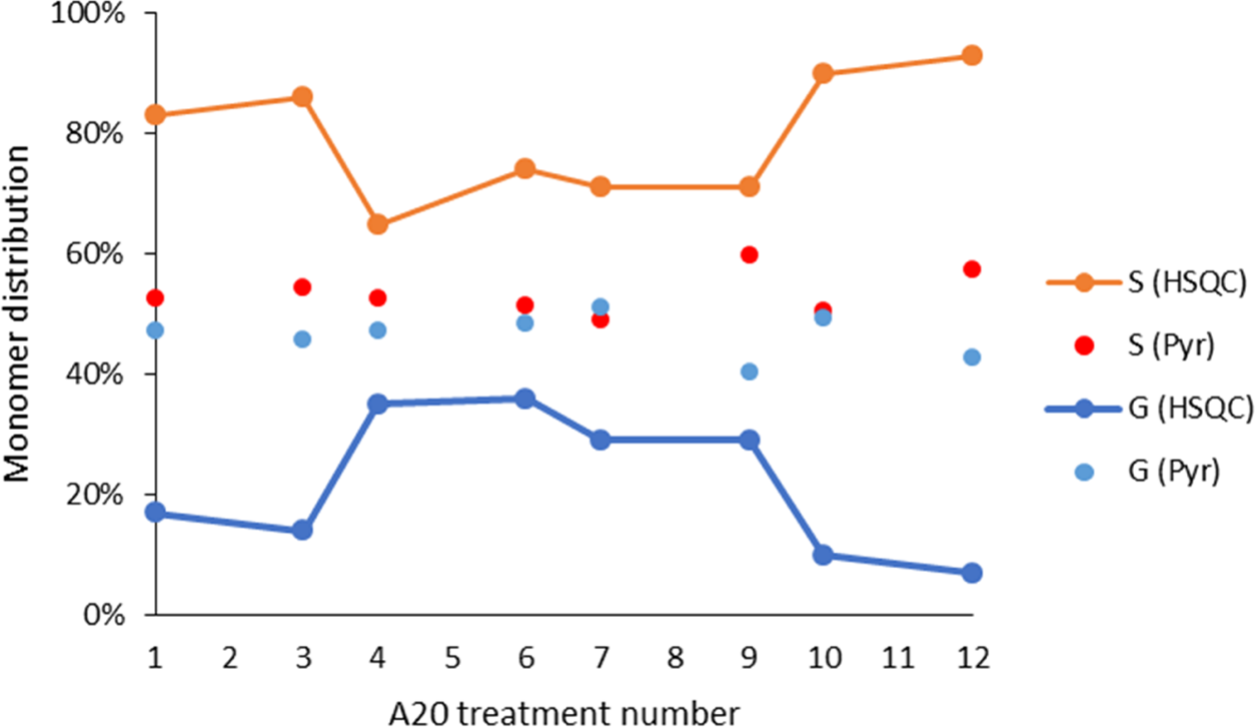
Lignin monomer ratios obtained through HSQC analyses and pyr-GC/MS.

Analyzing the S/G ratios measured via HSQC and pyr-GC/MS for the acid-catalyzed **A20** organosolv lignins (Figure 5), less established information is available for correlating the observed trends. Whereas the pyr-GC/MS data indicate a somewhat even distribution of the two monomers, the HSQC presents widely differing contents, especially for water content extremes.

Combined analyses suggest at this point that the extracted lignins are eventually enriched by furans as a function of the isolation conditions, with these furans eventually further condensing with acetone in the extraction solvent. The introduction of furans occurs with their decreasing content of functionality as the water content decreases. At the same time, there is a trend driving the lignin monomer distribution toward unity, which is observable through both HSQC and pyr-GC/MS.

**Figure 6:**
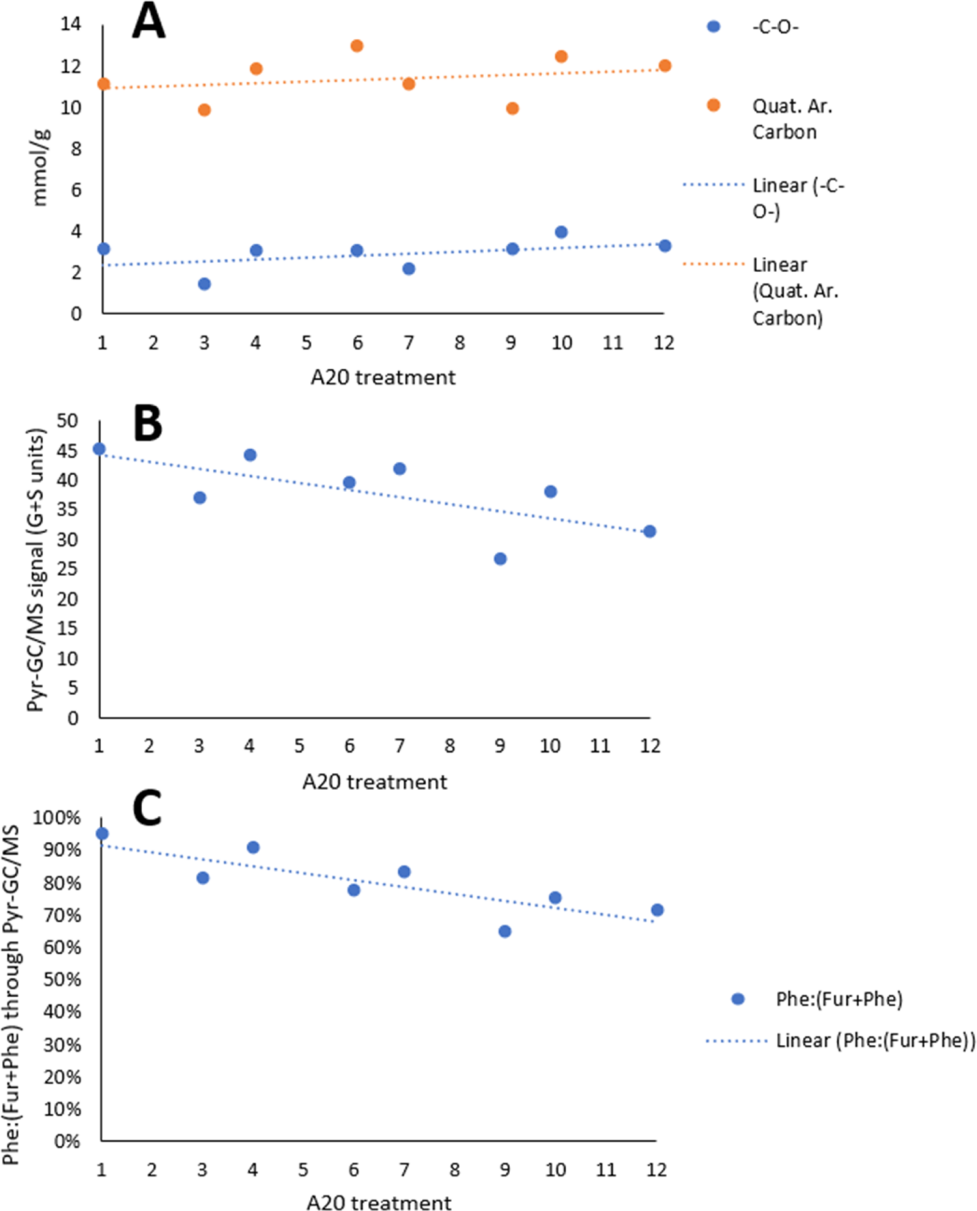
(A) Quantity of quaternary aromatic carbons (ArC-O) and aliphatic R-**C**-O-R groups as measured by quantitative ^13^C NMR analyses. (B) Combined intensities of G and S fragments as measured by pyr-GC/MS. (C) Mass ratio of phenolics to the combined phenolics+furans as measured by pyr-GC/MS.

The above discussion hints at the fact that the detailed structural description of **A20** lignins is eventually more complex, and thus, as a first approach, bulk properties and bond types will be employed to describe the overall situation. The HSQC shifts observed in the region downfield of the methoxy signal are integrated and interpreted as tertiary carbons present in ether linkages (57.0–67.0 ppm in the ^13^C NMR spectra). This is done since the region is in practice vacant of shifts belonging to traditional lignin motifs. Next, the content of aromatic quaternary carbons was measured through the ^13^C region between 140.0 and 160.0 ppm.^45^ Finally, these data were compared against the overall signal obtained for the two major lignin monomers obtained through pyr-GC/MS. The data obtained by this approach are graphically displayed in Figure 6A–C, respectively.

The signal delineated upon analysis by pyr-GC/MS swings in accordance with the overall content of phenolics found in the extracted lignins. In other words, they increase/decrease as a result of furan pollution, which in the pyr-GC/MS analysis is again influenced by the application of acetone during extraction. The fact that the previously unassigned signals, herein classified as R-C**-**O-R, largely follow the trend for the quaternary aromatic carbons suggests that these carbons indeed represent aryl–alkyl ether linkages. Next, both the quaternary carbons and C–O bond contents slowly increase as the water content is reduced. In an opposing trend, the release of monomeric units shows the same relative behavior between the respective treatments. Admittingly within the error ranges, one conspicuous difference between the points presented in Figure 4 can be identified for treatment **A20-6**: liberation of lignin monomeric units through pyr-GC/MS seems to be reduced. Also unique for this treatment is the occurrence of a signal in the ^13^C NMR spectrum between 48.50 and 50.25 ppm; the fact that this signal is absent in the HSQC suggests that it belongs to a quaternary carbon. The striking aspect of this is the elevated intensity, when interpreting it as a quaternary carbon linker forming as a consequence of **A20-6** treatment (Figure S1), in combination with, or in light of, the decreased release of lignin monomers during pyr-GC/MS for this lignin. Such quaternary linkages are reported to form through, for example, condensation of ketones in levulinic acid with phenolic rings in the presence of strong Brönsted mineral acids, such as H_2_SO_4_,^46^ generating a condensed structure likely to impede facile formation of volatiles upon pyrolysis. The very likely fact that a reduced water content leads more generally to an increase in condensed structures would require more investigation. Another source, which also could increase the aryl–ether content alongside that of R–C-O-R’ is obviously found in furan structures such as 5-HMF and its derivatives. However, considering the fact that the syringyl content appears to decrease when moving from acid-free to acid-catalyzed extraction, hence from production of **A0** lignins to **A20** lignins hints at the elevated reactivity of the S units,^47^ and thus the probable occurance of methoxy substitution reactions that have been reported before.^48^ Nonetheless, the formation of new aryl–aryl/alkyl ethers is suitable to at least partially explain the discrepancy between the measured content of methoxy groups and quaternary aromatic carbons linked to oxygens (Table 5). Thus, the reduced release of native lignin monomers can be argued to originate from both the presence of furan motifs and chemical alteration.

It has to be noted, as a note of caution, that some of the discussed structures, e.g., 4-(2-furanyl)-3-buten-2-one, eventually indicate the reactivity of acetone under certain treatment conditions. The structures can be seen as an aldol condensation product between furfural and acetone. Acetone is also reactive as an acetal-building element, and some cross-peaks in the HSQC could be interpreted as signs of an acetone-born acetal incorporating lignin β-*O*-4′ structures as diols. These aspects would need to be further elucidated.

### 3.3 Hemicellulose Characterization

The next fraction to be considered is the sugars and polysaccharides extracted in the aqueous phase. The compositions of the aqueous products for all the treatments investigated are detailed in Table 7, whereas the hemicellulose mass balances are presented in Tables S2.3.1 and S2.3.2.

**Table 7:** Sugar Composition of the Aqueous Product Obtained from the **A0** and **A20** Processing Series

compound	monomers [%m/m mon.+olig.]	cellulose monomers [g/100g_biomass_]	hemicell. monomers [g/100g_biomass_]	total monomers [g/100g_biomass_]	cellulose oligomers [g/100g_biomass_]	hemicell. oligomers [g/100g_biomass_]	total oligomers [g/100g_biomass_]	tot. cellulose sugars [g/100g_biomass_]	tot. hemicell. sugars [g/100g_biomass_]	dehydration products [g/100g_biomass_]
**Treatment**										
**A0-1**	30.18	0.82	1.65	2.48	0.00	5.73	5.73	0.82	7.38	2.14
**A0-2**	29.03	0.23	1.77	2.00	0.19	4.70	4.89	0.42	6.47	2.06
**A0-3**	19.10	0.16	1.41	1.57	0.32	6.31	6.63	0.48	7.72	2.24
**A0-4**	23.23	0.19	1.04	1.23	0.15	3.93	4.08	0.34	4.97	1.65
**A0-5**	21.19	0.17	1.02	1.20	0.22	4.23	4.45	0.39	5.25	2.00
**A0-6**	20.48	0.14	1.02	1.15	0.26	4.22	4.48	0.40	5.24	1.99
**A0-7**	37.91	0.60	0.70	1.30	0.00	2.14	2.14	0.60	2.84	1.43
**A0-8**	22.39	0.12	0.67	0.79	0.09	2.65	2.73	0.20	3.32	1.57
**A0-9**	20.74	0.00	0.70	0.70	0.17	2.52	2.69	0.17	3.23	1.87
**A0-10**	31.63	0.08	0.45	0.53	0.01	1.14	1.15	0.08	1.60	1.29
**A0-11**	37.01	0.01	0.61	0.62	0.07	0.98	1.05	0.09	1.58	0.47
**A0-12**	33.30	0.09	0.69	0.78	0.04	1.53	1,56	0.13	2.22	2.63
**A20-1**	91.07	3.86	5.35	9.22	0.90	0.00	0,9	4.76	5.35	4.79
**A20-2**	95.16	4.54	5.15	9.69	0.49	0.00	0,49	5.03	5.15	5.89
**A20-3**	92.84	5.04	3.75	8.80	0.68	0.00	0,68	5.72	3.75	7.70
**A20-4**	86.09	3.74	5.16	8.90	1.44	0.00	1,44	5.18	5.16	4.52
**A20-5**	91.14	3.81	4.86	8.68	0.84	0.00	0,84	4.66	4.86	5.71
**A20-6**	100.00	4.58	4.77	9.34	0.00	0.00	0.00	4.58	4.77	6.23
**A20-7**	84.43	3.25	5.53	8.79	1.69	0.00	1.69	4.94	5.53	4.00
**A20-8**	10.44	0.38	0.57	0.95	4.50	3.62	8.13	4.88	4.19	2.50
**A20-9**	82.01	0.63	0.49	1.11	0.24	0.00	0.24	0.87	0.49	3.90
**A20-10**	100.00	0.76	1.06	1.81	0.00	0.00	0.00	0.76	1.06	1.62
**A20-11**	100.00	2.28	2.61	4.89	0.00	0.00	0.00	2.28	2.61	2.90
**A20-12**	100.00	4.60	3.91	8.50	0.00	0.00	0.00	4.60	3.91	3.96

In the absence of mineral acid, the three highest water contents, i.e., 60, 50, and 40%v/v, are generally performing better when considering overall hemicellulosic sugar extraction into the aqueous phase. Despite this, the overall extraction efficiency is, however, relatively low, ranging from 1.58 to 7.38%m/m (4.71–32.30%m/m for hemicellulosic sugars in the raw material on a polymeric basis). The extraction of cellulosic species is in general negligible, as expected. Despite the contents being low, cellulose oligomers were extracted with increasing efficiencies within each set of samples using the same water content when the acetone content is increased, which bears similarities with decreasing cellulose pulp recoveries for the acid-catalyzed treatments. Considering the dehydration products of the **A0** series (Table S4.3), the overall contents are fairly low, reflecting the relatively mild conditions applied. This formation of condensation products correlates with the overall hemicellulosic extraction for which a high content of residual hemicellulose in the pulp yields lower formation of furans in the lignins.

The sugar compositions of the aqueous product obtained from the acid-catalyzed treatments are also listed in Table 7. When evaluating the monomers from cellulose and hemicellulose at different water contents, i.e., 60 and 50%v/v, cellulose and hemicellulose monomers follow opposing trends, with the former increasing while the latter decreases upon the addition of acetone. Looking at the formation of degradation products for these treatments (Table S4.4), the same trend is valid for the hexose sugars with the formation of a greater amount of 5-HMF upon partial replacement of ethanol with acetone. For a water content of 30%v/v, this trend is observed again, whereas at 40%v/v water, a drop in content of both hemicellulose and cellulosic sugar species (monomers and oligomers) appears while the degradation product contents increase readily upon acetone addition. Considering Figure 4, i.e., the phenol/(phenol + furan) ratio for treatments **A20-7** and **A20-9**, these treatment pairs, which show by far the largest drop (83 to 65%) in the phenol content due to furan “pollution”, alongside the increase in degradation products, seem indeed to favor the formation of sugar dehydration products. To further evaluate this, the total phenolic contents for the performed treatments were investigated (Tables S4.3 and S4.4). For the treatments employing acid, i.e., the **A20** series, total phenolics contents increase, as does the sum of furfural and 5-HMF, whereas in the nonacid catalyzed treatments this is not the case. This is in accordance with the increased hydrolytic activity of the system. The acetic acid contents for these apparently highly degradative conditions drop, since the same conditions, on the other hand, favor acid-catalyzed (trans-)esterifications.

**Figure 7:**
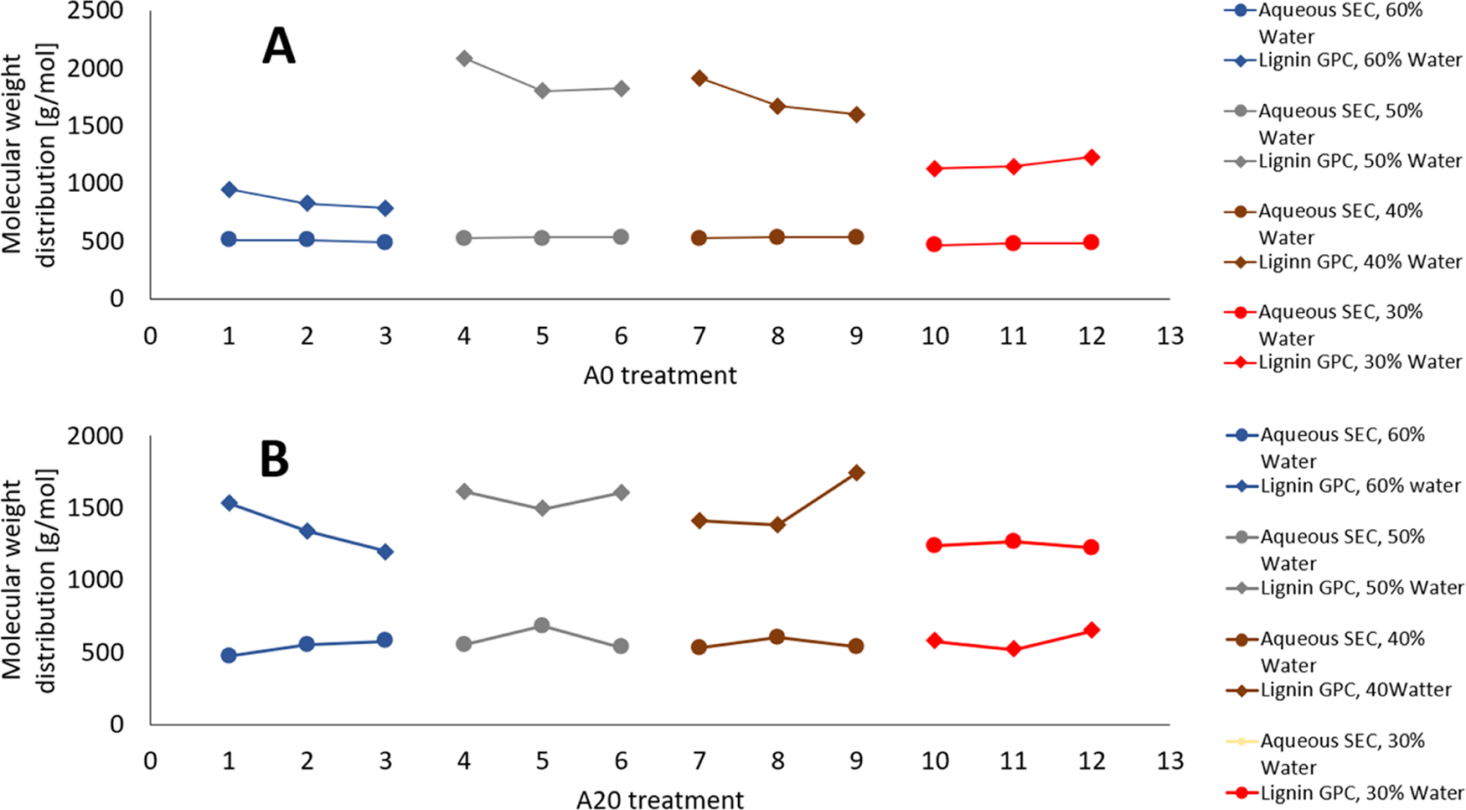
Comparison of molecular weights (Mn) obtained for the lignins and aqueous products of (A) nonacid catalyzed treatments (**A0** series) and (B) acid-catalyzed treatments (**A20** series).

As a final look into the characteristics of the sugar fraction obtained through the aqueous product, GPC analyses were performed to elucidate the distribution of molecular weights (Figure 5). While the variations are small, they are consistent with the fact that the majority of the aqueous product is consistently made out of larger polysaccharides (oligomeric) for the nonacid catalyzed system. This is not as readily deciphered when looking at the size distributions obtained from the acid-catalyzed treatments where there are greater variations in the size distributions of the aqueous product. The fact that most of the intact sugar moieties obtained in the aqueous product are of monomeric type (Table 6), whereas the measured molecular weights indicate that they in fact should be larger than what was measured for the nonacid catalyzed treatments, is noteworthy. Whereas the **A0** treatments seem to show a correlation between sugar products due to the relatively low severity, it would appear reasonable to consider that the aqueous product of the treatments employing 20 mm H_2_SO_4_, i.e., the **A20** samples, could carry dehydration structures. Looking also at the development of the molecular weight of the isolated lignins, it becomes obvious that these data reflect the extent to which the system has experienced depolymerization vs repolymerization events. Interestingly, the treatments performed with two additions of acetone (**A0-6** and **A0-9** in Figure 7A) present molecular weight distributions for which an increase in lignin molecular weight is also observed, whereas the aqueous products appear to be constituted by structures of lower molecular weight. This can be seen as another hint for the fact that the isolated lignins could thus comprise structures formed by condensation or “pollution” between furans and aromatics depleting the aqueous product and instead enriching the lignin fraction with larger moieties.

The gradual increase in dehydration products upon acetone addition is further indicated by the sum of furfural and 5-HMF measured in the aqueous products obtained from the acid-catalyzed treatments (Table S4.4), which, in general, correlate with increases in the total phenolic contents. This trend is not clear from the nonacid catalyzed treatment (Table S4.3), suggesting that the aromatic moieties instead mainly originate from native lignin.

Data eventually suggest that the extraction process and the reactions involved can be accelerated by the addition of acetone for specific water contents. For example, within the three treatments performed at 40%v/v water (**A20-7**, **A20-8**, and **A20-9**), fractionation appears to proceed significantly differently. Moving from **A20-7** to **A20-8**, there is an apparent increase in the oligomeric sugar concentration (Table 6), which is accompanied by an increase in the molecular weight measured through aqueous GPC. Moving from **A20-8** to **A20-9**, there is low detection of either sugar monomers or oligomers, whereas the lignin molecular weight increases. At the same time, there is an increase in both furfural and HMF contents. These findings, alongside the results obtained by quantified HSQC and pyr-GC/MS, thus indicate eventual synergistic effects in a range of water acetone mixtures. In fact, 60%v/v water appears to exist close to a critical threshold, which depending on acetone addition can be used to manipulate the properties of the extracted polysaccharides and their derivatives.

## 4 Conclusions

The data presented have been discussed in a holistic way in order to understand how the entire fractionation process is influenced by the conditions applied. A focus was placed also on understanding the effect of the “tweaking” achieved by the triple solvent system in connection to the application of an acid catalyst. Some general trends can be delineated: (1) Avoiding the application of a mineral acid during the organosolv process allows extraction of native lignins, for which a tuning of the molecular weight, and to some extent also of the dominating interunit motifs can be achieved by changing the solvent composition (**A0-1** to **A0-12**). (2) In the absence of an acid, the fractionation process experiences limitations due to either reduced lignin and or hemicellulose solubility/depolymerization (lignin solubility issues encountered for **A0-1** to **A0-3**, whereas hemicellulose remains in the matrix especially for **A0-10** to **A0-12**, respectively), independently of the use of a third solvent. (3) Applying an acid largely overcomes the aforementioned issues, revolving around extraction, where only limited lignin solubility at the highest water contents, in more pronounced form for **A20-1** to **A20-3**, to a lesser extent for **A20-4** to **A20-6**, limits the fractionation itself. (4) Replacing ethanol with acetone in the presence of acid redirects the product streams generated during the fractionation, and favors the formation of acetone–furan cross-products (**A20-3**, **6**, **9**, and **12**). (5) Formation of the distinct types of furans is followed by the formation of new quaternary aromatic carbons and R–C-O-R linkages alongside furan “pollution” in the lignin product, seen in increasing concentrations for **A20-1** to **A20-12**.

## Supporting Information Available

Additional tables containing various analytical data for the various fractions discussed throughout the manuscript (PDF)
