# Supplementary material for: Furan Distribution as a Severity Indicator upon Organosolv Fractionation of Hardwood Sawdust through a Novel Ternary Solvent System
Source: ACS Sustain Chem Eng. 2024 Jan 12;12(4):1666–80. doi: 10.1021/acssuschemeng.3c07236 (PMC10828987; doi:10.1021/acssuschemeng.3c07236)
Supplement: Supplementary file 1 — sc3c07236_si_001.pdf [file sc3c07236_si_001.pdf]

Supplementary information

Furan distribution as severity indicator upon organosolv fractionation of hardwood sawdust through novel ternary solvent system

Petter Paulsen Thoresen,[a] Irene Delgado Vellosillo,[a] Heiko Lange,[a,b,c] Ulrika Rova,[a] Paul Christakopoulos[a] and Leonidas Matsakas[a,]*

[a] Biochemical Process Engineering, Division of Chemical Engineering, Department of Civil, Environmental and Natural Resources Engineering, Luleå University of Technology, SE-971 87 Luleå, Sweden

[b] Department of Earth and Environmental Sciences, University of Milano-Bicocca, Piazza della Scienza 1, 20126 Milan, Italy

[c] NBFC – National Biodiversity Future Center, 90133 Palermo, Italy

*Corresponding author: Leonidas Matsakas, Department of Civil, Environmental and Natural Resources Engineering, SE-971 87 Luleå Sweden, leonidas.matsakas@ltu.se, tel.: +46 (0) 920 493043

A. Number of pages: 14 (S1-S14)

B. Number of figures: 2

C. Number of Tables: 15

1: Page S1: Cover

2: Page S2: Lignin characterization. Tables S1.1-S1.2

3: Page S3: Lignin mass balances. Tables S2.1.1-S2.1.2

4: Page S4: Cellulose mass balances. Tables S2.2.1-S2.2.2

5: Page S5: Hemicellulose mass balance. Tables S2.3.1-S2.3.2

6: Page S6: Quaternary carbon content. Figure 1.

7: Page S7-S11: Prominent figures and tables discussed in manuscript. S4.1 (lignin motifs obtained from pyr-GC/MS), S4.2 (lignin motifs obtained from pyr-GC/MS), S4.3 (dehydration products in liquor), and S4.4 (dehydration products in liquor). Figure S2 (lignin monomeric ratios from HSQC and Pyr-GC/MS).

8: Page S12: Table S5.1 (assigned NMR shifts).

9: Page S13 – page S28: Figures S2 – S18, detailing 13C NMR and HSQC spectra.

S1 Lignin characterization

Table S1.1: Compositional data for the lignins isolated without additional of mineral acid.

**Treatment**	**Klason lignin content [%g/g] Primary (wash)**	**Hemicellulosic sugar content [%g/g] Primary (wash)**	**Cellulosic sugar content [%g/g] Primary (wash)**
**A0-1**	0.85 (0.91)	2.01 (1.17)	0.29 (0.01)
**A0-2**	0.87 (0.89)	2.15 (1.17)	0.00 (0.01)
**A0-3**	0.90 (0.76)	2.03 (1.45)	0.24 (0.34)
**A0-4**	0.86 (0.89)	2.33 (1.03)	0.45 (0.11)
**A0-5**	0.89 (0.89)	1.68 (1.08)	0.44 (0.20)
**A0-6**	0.89 (0.90)	1.81 (0.00)	0.26 (0.00)
**A0-7**	0.87 (0.89)	2.68 (1.64)	0.43 (0.68)
**A0-8**	0.83 (0.89)	3.47 (1.53)	0.46 (0.54)
**A0-9**	0.88 (0.90)	2.32 (1.66)	0.37 (0.87)
**A0-10**	0.87 (0.88)	1.96 (1.30)	0.58 (1.08)
**A0-11**	0.90 (0.91)	1.42 (1.06)	0.36 (0.62)
**A0-12**	0.88 (0.90)	1.54 (1.18)	0.34 (0.48)

Table S1.2: Compositional data for the lignins isolated with additional of mineral acid.

**Treatment**	**Klason lignin content [%g/g] Primary (wash)**	**Hemicellulosic sugar content [%g/g] Primary (wash)**	**Cellulosic sugar content [%g/g] Primary (wash)**
**A20-1**	0.92 (0.89)	0.40 (0.38)	0.84 (0.71)
**A20-2**	0.92 (0.93)	0.24 (0.23)	0.53 (0.51)
**A20-3**	0.92 (0.91)	0.13 (0.22)	0.61 (0.63)
**A20-4**	0.92 (0.90)	0.19 (1.01)	0.57 (3.60)
**A20-5**	0.92 (0.91)	0.28 (0.77)	0.81 (1.65)
**A20-6**	0.91 (0.91)	0.22 (0.19)	0.59 (0.52)
**A20-7**	0.89 (0.86)	0.44 (1.37)	0.98 (2.64)
**A20-8**	0.91 (0.89)	0.24 (0.22)	0.57 (0.71)
**A20-9**	0.91 (0.90)	0.51 (0.12)	1.24 (2.84)
**A20-10**	0.82 (0.89)	0.50 (0.94)	1.58 (2.05)
**A20-11**	0.92 (0.86)	0.21 (0.50)	0.51 (7.94)
**A20-12**	0.92 (0.91)	0.27 (0.70)	0.90 (2.41)

S2 Mass balances

S2.1 Lignin mass balance

Table S2.1.1: Mass balance data for the lignins (Klason lignin content) isolated without addition of mineral acid.

Treatment	Lignin recovered in pulp [%g/g in raw]	Primary lignin recovered [%g/g in raw]	Wash lignin recovered [%g/g in raw]	Lignin recovered in primary liquid [%g/g in raw]	Lignin recovered in wash liquid [%g/g in raw]	Sum [%]
**A0-1**	59.26	37.08	4.50	6.64	2.01	109.49
**A0-2**	53.29	35.21	6.22	7.29	2.09	104.10
**A0-3**	50.30	44.09	5.52	7.91	11.61	119.43
**A0-4**	39.29	51.01	6.81	7.70	1.91	106.72
**A0-5**	35.37	51.29	7.34	6.56	2.09	102.65
**A0-6**	42.40	55.80	7.16	6.02	1.72	113.11
**A0-7**	44.80	44.40	6.46	3.71	0.79	100.16
**A0-8**	42.59	55.72	6.27	6.57	5.83	116.99
**A0-9**	42.03	49.28	4.96	5.02	0.59	101.87
**A0-10**	57.29	31.91	6.01	3.55	2.19	100.95
**A0-11**	60.40	39.71	3.08	4.45	0.74	108.39
**A0-12**	58.41	43.04	3.13	7.88	0.55	113.02

Table S2.1.2: Mass balance data for the lignins (Klason lignin content) isolated with addition of mineral acid.

Treatment	Lignin recovered in pulp [%g/g in raw]	Primary lignin recovered [%g/g in raw]	Wash lignin recovered [%g/g in raw]	Lignin recovered in primary liquid [%g/g in raw]	Lignin recovered in wash liquid [%g/g in raw]	Sum [%]
**A20-1**	38.38	44.07	6.64	6.34	0.64	96.07
**A20-2**	36.18	47.14	6.79	7.39	0.88	98.38
**A20-3**	37.38	52.97	7.64	7.92	0.35	106.26
**A20-4**	19.38	68.77	9.06	7.52	0.41	105.14
**A20-5**	15.10	67.38	9.30	7.62	0.37	99.76
**A20-6**	12.68	76.28	11.21	9.98	0.25	110.40
**A20-7**	7.44	76.43	10.07	7.18	0.42	101.54
**A20-8**	5.77	89.35	9.33	9.01	0.56	114.01
**A20-9**	5.88	98.56	11.04	12.05	0.58	128.11
**A20-10**	6.86	70.02	5.13	9.73	0.58	92.32
**A20-11**	5.53	81.59	9.60	10.60	0.35	107.66
**A20-12**	5.23	100.71	5.56	14.91	0.50	126.90

S2.2 Cellulose mass balance

Table S2.2.1: Mass balance data for the cellulose isolated without addition of mineral acid.

Treatment	Cellulose recovered in pulp [%g/g in raw]	Cellulose recovered in primary lignin [%g/g in raw]	Cellulose recovered in wash lignin [%g/g in raw]	Cellulose recovered in prim. Liq. as mono. [%g/g in raw]	Cellulose recovered in prim liq. as polym. [%g/g in raw]	Cellulose recovered in wash Liq. as mono. [%g/g in raw]	Cellulose recovered in wash liq. as polym. [%g/g in raw]	Sum [%g/g in raw]
**A0-1**	95.22	0.08	0.00	1.99	0.00	0.03	0.17	97.50
**A0-2**	83.90	0.00	0.00	0.56	0.58	0.00	0.22	85.26
**A0-3**	101.15	0.07	0.01	0.38	0.97	0.07	0.20	102.86
**A0-4**	92.89	0.16	0.01	0.47	0.44	0.00	0.22	94.19
**A0-5**	92.21	0.16	0.01	0.42	0.66	0.00	0.29	93.74
**A0-6**	98.52	0.10	0.00	0.33	0.78	0.00	0.21	99.94
**A0-7**	102.07	0.13	0.03	1.46	0.00	0.00	0.14	103.84
**A0-8**	95.43	0.18	0.02	0.29	0.26	0.00	0.13	96.31
**A0-9**	99.41	0.12	0.03	0.00	0.51	0.00	0.13	100.20
**A0-10**	103.91	0.13	0.04	0.19	0.02	0.07	0.00	104.35
**A0-11**	110.02	0.10	0.01	0.03	0.22	0.27	0.00	110.65
**A0-12**	107.16	0.10	0.01	0.22	0.11	0.00	0.06	107.67

Table S2.2.2: Mass balance data for the cellulose isolated with addition of mineral acid.

Treatment	Cellulose recovered in pulp [%g/g in raw]	Cellulose recovered in primary lignin [%g/g in raw]	Cellulose recovered in wash lignin [%g/g in raw]	Cellulose recovered in prim. Liq. as mono. [%g/g in raw]	Cellulose recovered in prim liq. as polym. [%g/g in raw]	Cellulose recovered in wash Liq. as mono. [%g/g in raw]	Cellulose recovered in wash liq. as polym. [%g/g in raw]	Sum [%g/g in raw]
**A20-1**	81.46	0.24	0.03	9.34	2.70	1.77	0.56	96.11
**A20-2**	75.86	0.16	0.02	10.99	1.47	2.20	0.37	91.08
**A20-3**	75.77	0.21	0.03	12.21	2.03	1.56	0.00	91.80
**A20-4**	83.31	0.26	0.22	9.06	4.30	1.61	0.79	99.50
**A20-5**	74.61	0.36	0.10	9.23	2.52	1.68	0.37	88.86
**A20-6**	72.62	0.30	0.04	11.07	0.00	2.09	14.06	100.19
**A20-7**	73.17	0.51	0.19	7.87	5.05	0.07	2.49	89.35
**A20-8**	66.04	0.34	0.05	0.91	13.45	0.12	2.27	83.18
**A20-9**	59.76	0.81	0.21	1.52	0.73	0.56	0.21	63.79
**A20-10**	68.17	0.82	0.07	1.83	0.00	0.29	2.70	73.89
**A20-11**	62.49	0.27	0.53	5.52	0.00	1.60	0.00	70.42
**A20-12**	51.92	0.60	0.09	11.12	0.00	1.99	0.00	65.72

S2.3 Hemicellulose mass balance

Table S2.3.1: Mass balance data for the hemicellulose isolated without addition of mineral acid.

Treatment	Hemicell. recovered in pulp [%g/g in raw]	Hemicell. recovered in primary lignin [%g/g in raw]	Hemicell. recovered in wash lignin [%g/g in raw]	Hemicell. recovered in prim. Liq. as mono. [%g/g in raw]	Hemicell. recovered in prim liq. as polym. [%g/g in raw]	Hemicell. recovered in wash Liq. as mono. [%g/g in raw]	Hemicell. recovered in wash liq. as polym. [%g/g in raw]	Sum [%g/g in raw]
**A0-1**	30.51	0.84	0.06	7.03	29.75	1.01	4.30	73.49
**A0-2**	35.46	0.83	0.08	7.54	24.96	5.85	0.00	74.72
**A0-3**	30.43	0.96	0.12	5.99	32.30	1.16	5.30	76.26
**A0-4**	48.02	1.32	0.08	4.43	20.18	1.20	3.55	78.78
**A0-5**	47.31	1.09	0.10	4.35	21.45	0.99	4.34	79.63
**A0-6**	58.80	1.30	0.00	4.32	21.93	0.68	3.17	90.21
**A0-7**	67.59	1.56	0.13	2.98	10.32	0.70	2.74	86.03
**A0-8**	66.86	2.55	0.12	2.85	13.19	0.54	2.44	88.55
**A0-9**	65.64	1.45	0.09	3.00	12.66	0.52	2.20	85.55
**A0-10**	89.23	0.82	0.11	1.93	5.44	1.63	0.00	99.15
**A0-11**	95.82	0.84	0.04	2.58	4.71	2.11	0.00	106.12
**A0-12**	86.22	0.96	0.05	2.93	7.33	0.00	1.21	98.71

Table S2.3.2: Mass balance data for the hemicellulose isolated with addition of mineral acid.

Treatment	Hemicell. recovered in pulp [%g/g in raw]	Hemicell. recovered in primary lignin [%g/g in raw]	Hemicell. recovered in wash lignin [%g/g in raw]	Hemicell. recovered in prim. Liq. as mono. [%g/g in raw]	Hemicell. recovered in prim liq. as polym. [%g/g in raw]	Hemicell. recovered in wash Liq. as mono. [%g/g in raw]	Hemicell. recovered in wash liq. as polym. [%g/g in raw]	Sum [%g/g in raw]
**A20-1**	0.00	0.26	0.04	22.79	0.10	6.51	0.00	29.69
**A20-2**	0.00	0.16	0.02	21.92	0.04	5.96	21.74	49.84
**A20-3**	0.00	0.07	0.02	15.98	0.12	3.62	0.00	19.81
**A20-4**	0.00	0.13	0.10	21.94	0.00	5.75	0.00	27.92
**A20-5**	0.00	0.32	0.10	20.69	0.26	5.61	0.00	26.98
**A20-6**	0.00	0.32	0.02	20.29	0.00	5.50	16.65	42.78
**A20-7**	0.00	0.36	0.21	23.54	0.60	1.10	4.15	29.96
**A20-8**	0.00	0.39	0.02	2.42	18.38	0.41	2.50	24.12
**A20-9**	0.00	0.52	0.18	2.06	0.15	1.33	0.00	4.25
**A20-10**	0.00	0.61	0.07	4.49	0.12	1.32	3.37	9.98
**A20-11**	0.00	0.21	0.07	11.09	0.57	5.68	0.00	17.63
**A20-12**	0.00	0.28	0.06	16.62	0.32	6.21	0.00	23.49

S3 Quaternary carbon content

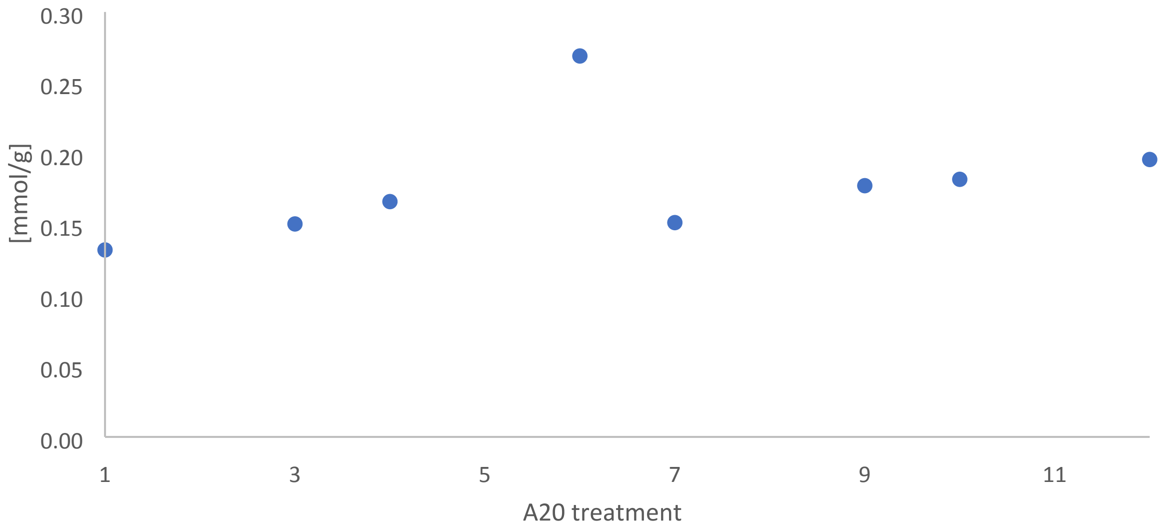

Figure S1. *Content of quaternary carbon linker (48.5-50.25 ppm) for the various A20 treatments.*

S4 Prominent Figures and Tables discussed in manuscript

Table S4.1: Pyr-GC/MS data for selected lignins isolated subsequent acid-free organosolv processing. *N.B.*: names as listed in the NIST database have been used.

Identified compound	Content in samples [% area]							
	A0-1	A0-3	A0-4	A0-6	A0-7	A0-9	A0-10	A0-12
**Furfural**	ND	ND	ND	ND	ND	ND	ND	ND
**5-HMF**	ND	ND	ND	ND	ND	ND	ND	ND
**Furan, 2-(2-propenyl)-**	ND	ND	ND	ND	ND	ND	ND	ND
**2-Furancarboxaldehyde, 5-methyl-**	ND	ND	ND	ND	ND	ND	ND	ND
**3-Buten-2-one, 4-(2-furanyl)-**	ND	0.20	ND	0.21	ND	0.19	ND	0.20
**1-Heptanone, 1-(2-furanyl)-**	ND	ND	ND	ND	ND	ND	ND	ND
**Benzene, 1,2,3,4-tetramethoxy-5-(2-propenyl)-**	1.31	0.84	0.51	0.25	0.59	ND	0.82	ND
**alpha-*D*-Glucopyranose, 1,6-anhydro**	ND	ND	ND	ND	ND	ND	ND	ND
**Eugenol (G)**	0.96	0.80	0.71	0.77	1.00	0.80	0.97	0.98
**trans-Isoeugenol (G)**	2.13	2.14	1.54	2.26	2.02	2.00	2.29	2.47
**Vanillin (G)**	2.42	2.53	3.97	3.19	2.70	2.94	3.78	3.42
**Apocynin (G)**	1.65	1.89	1.49	1.90	1.37	1.63	1.77	1.86
**Benzenepropanol, 4-hydroxy-3-methoxy- (G)**	0.69	0.64	0.76	0.63	ND	ND	0.55	ND
**Butyrovanillone (G)**	ND	ND	ND	ND	ND	ND	ND	ND
**Phenol, 2-methoxy-4-(1-propenyl)- (G)**	ND	ND	ND	ND	ND	ND	ND	ND
**Phenol, 2,6-dimethoxy- (G)**	4.77	4.87	4.00	4.82	3.38	3.88	4.45	4.66
**2-Methoxy-4-vinylphenol (G)**	3.94	3.48	2.81	3.41	3.36	3.50	3.42	3.71
**Phenol, 4-ethyl-2-methoxy-**	1.69	1.63	1.60	1.93	1.59	1.64	1.81	1.78

**(G)**								
**Phenol, 2-methoxy- (G)**	2.11	2.08	1.83	2.37	2.09	2.07	2.32	2.45
**trans-Sinapyl alcohol (S)**	3.12	3.44	2.49	0.99	1.49	1.30	2.01	1.79
**(E)-2,6-Dimethoxy-4-(prop-1-en-1-yl)phenol (S)**	1.64	1.60	0.95	1.47	1.19	0.95	1.10	1.16
**Butylsyringone (S)**	ND	0.63	ND	0.57	ND	ND	0.60	0.52
**Phenol, 4-ethenyl-2,6-dimethoxy- (S)**	4.32	5.23	4.40	4.54	3.30	3.96	4.18	4.19
**3,5-Dimethoxy-4-hydroxytoluene (S)**	4.16	4.32	2.91	4.22	3.05	2.80	3.01	3.30
**Phenol, 2,6-dimethoxy-4-(2-propenyl)- (S)**	2.57	2.76	2.01	2.50	1.87	1.72	2.07	2.07
**(E)-2,6-Dimethoxy-4-(prop-1-en-1-yl)phenol (S)**	5.70	5.88	4.79	5.45	3.89	3.87	4.46	4.58
**Benzaldehyde, 4-hydroxy-3,5-dimethoxy- (S)**	5.93	6.85	8.20	6.81	4.27	4.47	6.42	5.76
**Ethanone, 1-(4-hydroxy-3,5-dimethoxyphenyl)- (S)**	3.44	3.57	2.82	3.19	2.21	2.27	2.80	2.70
**Syringylacetone (S)**	0.88	0.94	0.92	0.93	0.73	2.97	1.09	0.98
**1-Propanone, 1-(4-hydroxy-3,5-dimethoxyphenyl)- (S)**	1.11	1.29	0.74	1.13	0.83	0.78	0.89	0.85
**Creosol**	3.17	3.01	2.37	3.25	2.68	2.85	2.55	3.01
**Methylated (2/3) phenol**	1.54	1.48	1.54	1.93	1.65	1.66	1.61	1.84
**Phenol, 4-propyl-**	ND	ND	ND	ND	ND	ND	ND	ND
**Formic acid, 2,3-dimethylphenyl ester**	0.54	0.51	ND	0.68	ND	0.93	0.69	ND
**(G:S:H)**	(35:62:3)	(33:65:3)	(35:62:3)	(37:60:4)	(41:55:4)	(39:57:4)	(39:58:3)	(40:57:4)

ND: indicates that a compound is below detection limit of %area = 0.5.

%area: Corresponding area for a compound relative to the sum of all the signal areas in the chromatogram.

Table S4.2: Pyr-GC/MS data for selected lignins isolated from acid-catalyzed organosolv processing. *N.B.*: names as listed in the NIST database have been used.

**Identified compound**	**Content in samples [%A]**							
	A20-1	A20-3	A20-4	A20-6	A20-7	A20-9	A20-10	A20-12
**Furfural**	2.29	2.98	2.91	4.97	4.43	4.91	5.36	3.71
**5-HMF**	0.79	1.09	1.66	2.01	4.35	4.08	7.30	4.31
**Furan, 2-(2-propenyl)-**	ND	1.26	ND	1.10	ND	1.62	ND	1.27
**2-Furancarboxaldehyde, 5-methyl-**	ND	0.76	0.57	0.79	1.16	1.48	1.68	0.94
**3-Buten-2-one, 4-(2-furanyl)-**	ND	3.99	ND	4.09	ND	5.46	ND	3.80
**1-Heptanone, 1-(2-furanyl)-**	ND	ND	ND	ND	ND	ND	0.27	ND
**4,5,6-Trimethoxy-7-methyl-3H-2-benzofuran-1-one; Benzene, 1,2,3,4-tetramethoxy-5-(2-propenyl)-**	ND	ND	ND	ND	ND	ND	ND	ND
**alpha-D-Glucopyranose, 1,6-anhydro**	0.63	ND	ND	ND	0.77	ND	0.74	0.98
**Eugenol (G)**	0.76	0.54	0.74	0.64	0.65	ND	ND	ND
**trans-Isoeugenol (G)**	ND	ND	ND	ND	ND	ND	ND	ND
**Vanillin (G)**	2.47	2.27	2.56	3.06	2.11	1.62	2.09	1.74
**Apocynin (G)**	1.94	1.94	1.73	2.27	1.56	1.43	1.45	1.30
**Benzenepropanol, 4-hydroxy-3-methoxy- (G)**	0.82	0.56	0.51	0.56	0.54	0.51	ND	1.12
**Butyrovanillone (G)**	1.64	1.44	2.08	2.04	2.28	1.09	2.46	1.51
**Phenol, 2-methoxy-4-(1-propenyl)- (G)**	0.66	0.58	0.58	0.54	0.78	0.53	0.74	0.53
**Phenol, 2,6-dimethoxy- (G)**	5.86	4.59	5.85	4.27	5.05	3.29	4.71	3.19
**2-Methoxy-4-vinylphenol (G)**	3.04	1.69	3.09	1.87	2.52	1.40	2.26	1.34
**Phenol, 4-ethyl-2-methoxy- (G)**	1.77	1.57	1.92	1.44	1.88	1.07	1.72	0.94
**Phenol, 2-methoxy- (G)**	3.58	2.81	3.37	2.73	3.73	2.53	3.29	2.21
***trans*-Sinapyl alcohol (S)**	ND	ND	ND	ND	ND	ND	ND	ND
**(*E*)-2,6-Dimethoxy-4-(prop-1-en-1-yl)phenol (S)**	ND	ND	ND	ND	ND	ND	ND	ND
**Butylsyringone (S)**	1.30	1.24	1.52	1.40	1.44	0.93	1.46	1.24
**Phenol, 4-ethenyl-2,6-dimethoxy- (S)**	2.11	1.84	1.74	1.79	1.53	1.19	1.34	1.18
**3,5-Dimethoxy-4-hydroxytoluene (S)**	4.79	3.97	4.19	4.07	4.36	3.13	4.35	2.75
**Phenol, 2,6-dimethoxy-4-(2-propenyl)- (S)**	0.71	0.73	0.85	0.81	0.74	0.61	0.78	0.61
**(*E*)-2,6-Dimethoxy-4-(prop-1-en-1-yl)phenol (S)**	1.98	1.27	1.21	1.21	1.50	0.99	1.38	1.24
**Benzaldehyde, 4-hydroxy-3,5-dimethoxy- (S)**	2.51	2.57	2.94	2.86	2.15	1.70	1.98	2.45
**Ethanone, 1-(4-hydroxy-3,5-dimethoxyphenyl)- (S)**	3.40	2.39	3.27	2.35	2.05	2.11	1.65	2.42
**Syringylacetone (S)**	1.35	1.29	1.48	1.31	2.01	1.76	2.10	1.98

**1-Propanone, 1-(4-hydroxy-3,5-dimethoxyphenyl)- (S)**	0.88	0.72	0.57	0.75	0.64	0.57	0.58	0.82
**Creosol**	4.52	3.39	4.32	3.82	5.15	0.37	4.86	2.58
**Methylated (2/3) phenol**	2.30	2.55	2.40	1.34	2.68	2.26	2.68	1.23
**Phenol, 4-propyl-**	ND	ND	ND	1.18	ND	1.43	ND	0.97
**Formic acid, 2,3-dimethylphenyl ester**	ND	0.59	ND	ND	0.57	0.38	ND	ND
**(G:S:H)**	(45:50:5)	(43:51:6)	(45:50:5)	(47:50:3)	(48:46:6)	(37:55:8)	(46:47:7)	(41:55:4)

ND: indicates that a compound is below detection limit of %area = 0.5.

%area: Corresponding area for a compound relative to the sum of all the signal areas in the chromatogram.

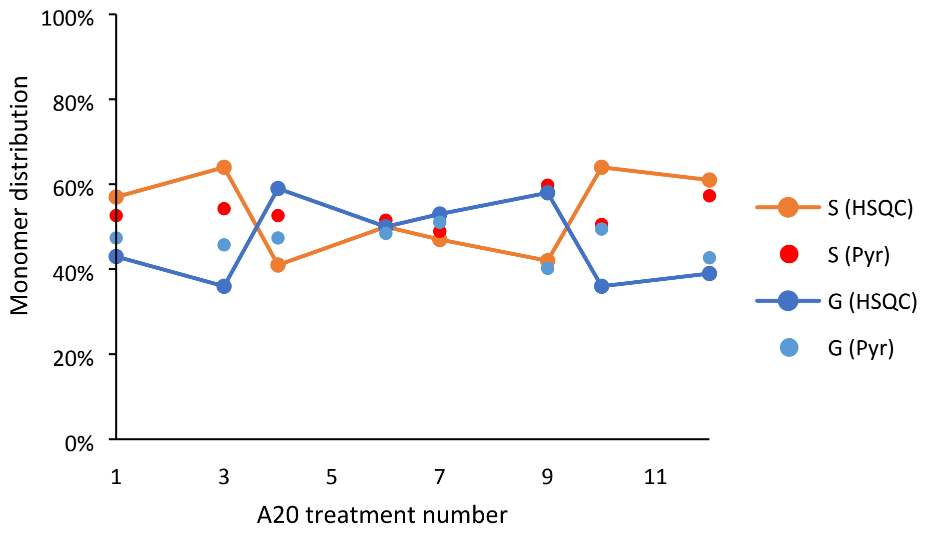

Figure S2. Lignin monomer ratios obtained through HSQC analyses and pyr-GC/MS.

Table S4.3: Dehydration products measured in the liquors isolated from non-acid catalyzed treatments.

Treatment	Furfural [g/100g$_{biomass}$]	HMF [g/100g$_{biomass}$]	Formic acid [g/100g$_{biomass}$]	Acetic acid [g/100g$_{biomass}$]	Tot. Phenolics [g/100g$_{biomass}$]
**A0-1**	0.10	0.06	0.00	1.89	0.09
**A0-2**	0.07	0.05	0.23	1.62	0.09
**A0-3**	0.07	0.05	0.39	1.65	0.08
**A0-4**	0.04	0.04	0.08	1.42	0.07
**A0-5**	0.05	0.04	0.12	1.71	0.08
**A0-6**	0.05	0.03	0.21	1.61	0.09
**A0-7**	0.03	0.02	0.14	1.18	0.06
**A0-8**	0.02	0.02	0.28	1.19	0.06
**A0-9**	0.03	0.02	0.41	1.33	0.08
**A0-10**	0.02	0.02	0.15	1.03	0.07
**A0-11**	0.02	0.02	0.18	0.17	0.08
**A0-12**	0.02	0.02	0.28	2.20	0.11

[a]Levulinic acid was not detected.

Table S4.4: Dehydration products measured in the liquors isolated from acid catalyzed treatments.

Treatment	Furfural [g/100g$_{biomass}$]	HMF [g/100g$_{biomass}$]	Formic acid [g/100g$_{biomass}$]	Acetic acid [g/100g$_{biomass}$]	Tot. Phenolics [g/100g$_{biomass}$]
**A20-1**	0.76	0.70	0.47	2.73	0.13
**A20-2**	1.02	0.69	0.91	3.14	0.13
**A20-3**	1.13	1.03	1.72	3.69	0.13
**A20-4**	0.65	0.66	0.71	2.40	0.10
**A20-5**	0.93	0.83	1.18	2.64	0.13
**A20-6**	0.72	0.89	1.41	3.09	0.12
**A20-7**	0.69	0.57	0.85	1.80	0.09
**A20-8**	0.94	0.93	0.35	0.16	0.12
**A20-9**	1.17	1.56	0.68	0.36	0.13
**A20-10**	0.20	1.10	0.00	0.28	0.04
**A20-11**	0.88	0.88	0.46	0.58	0.10
**A20-12**	0.93	1.25	0.33	1.34	0.11

[a]Levulinic acid was not detected.

S5 Assigned NMR shifts

Table S5.1: Shifts assigned for NMR analysis.

Motif	^{13}C	^{1}H
**G_2**[1]	110.15	6.93
**G_6**[1]	119.05-120.76	6.53-6.78
**$S_{2,6}$**	103.42	6.62
**-OMe**	56.5-55.0	-
**Quat. Phe C-OMe**	148.5-145.0	-
	151.0-153.5	-
**β-O-4' to G**		
**(C_α)**	71.29	4.91
**(C_β)**	83.21	4.34
**(C_γ)**	59.91	3.24
**β-O-4' to S**		
**(C_α)**	72.06	4.89
**(C_β)**	85.92	4.13
**(C_γ)**	59.43	3.41
**β-β'**		
**(C_α)**	87.03	5.44
**(C_β)**	53.05	3.48
**(C_γ)**	62.97	3.63
**β-5'**		
**(C_α)**	85.16	4.63
**(C_β)**	53.52	3.07
**(C_γ)**	70.88/71.05	4.19/3.81
**β-D-Xylopyr.**		
**(C_1)**	101.83	4.29
**(C_2)**	72.51	3.06
**(C_3)**	73.95	3.27
**(C_4)**	75.42	3.53
**(C_5)**	64.52	3.45
**BE**	80.87	4.59
**Furan C_4 type 1**	106.38	7.28
**Furan C_4 type 2**	122.86	7.53
**Furan C_3 type 2**[1]	112.75	6.77
**Furan C_3 type 1**	106.07	6.51
**Ketone**	207.2-206.5	-
**Ester**	170.5-169.0	-
**Furan aldehyde**	178.5-177.9	-

[1]A20 lignins show expanded guaiacyl shifts. Full range given for G_6 whereas G_2 overlaps with Furan C_3 type 2.

Spectroscopic data

A0-1

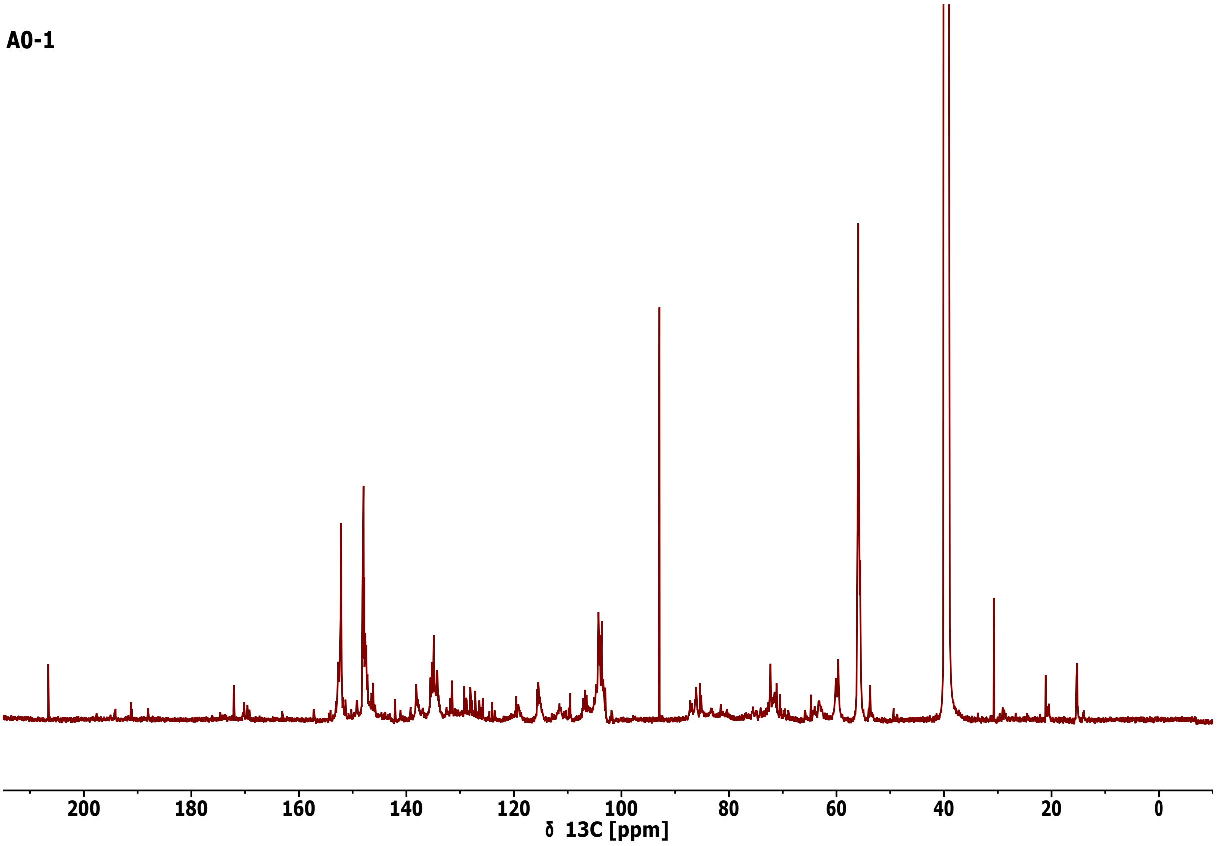

A0-1

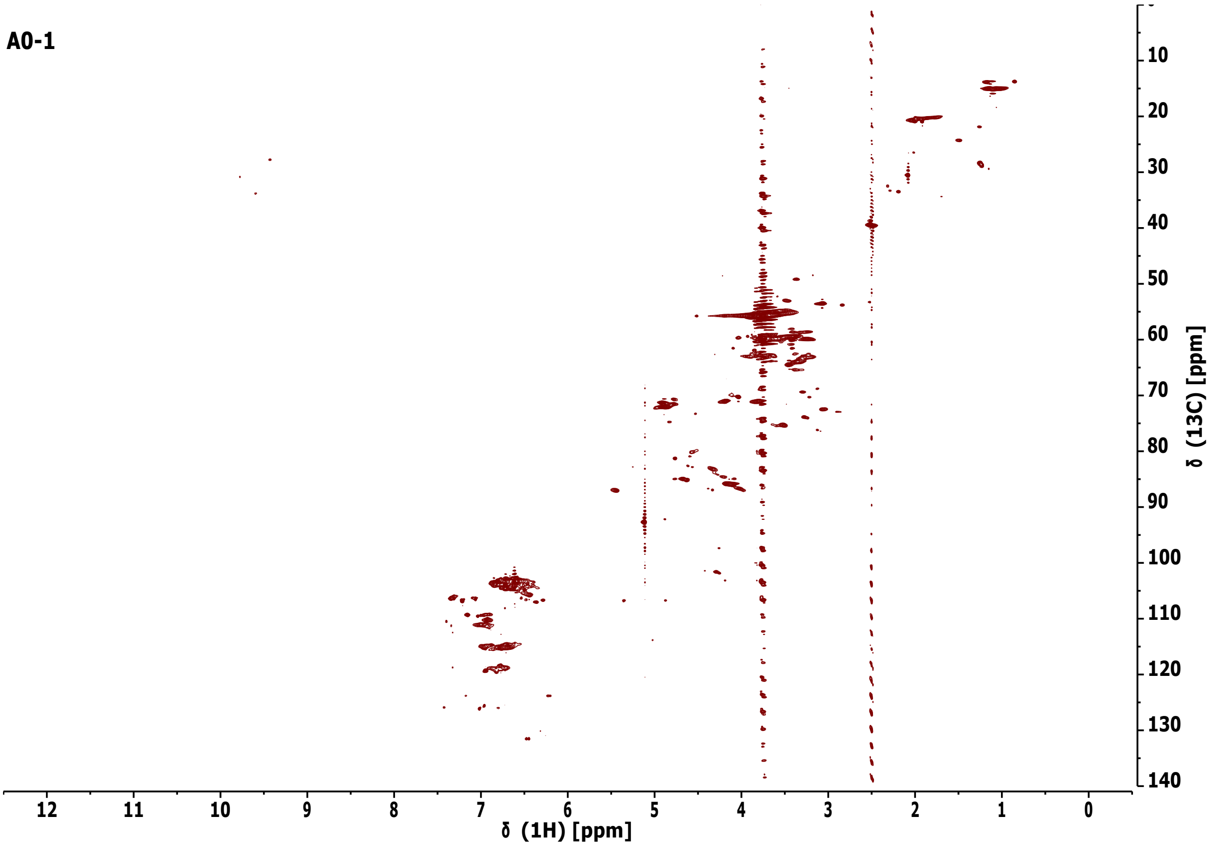

Figure S3. The ^{13}C (top) and HSQC (bottom) spectra for lignin sample **A0-1**.

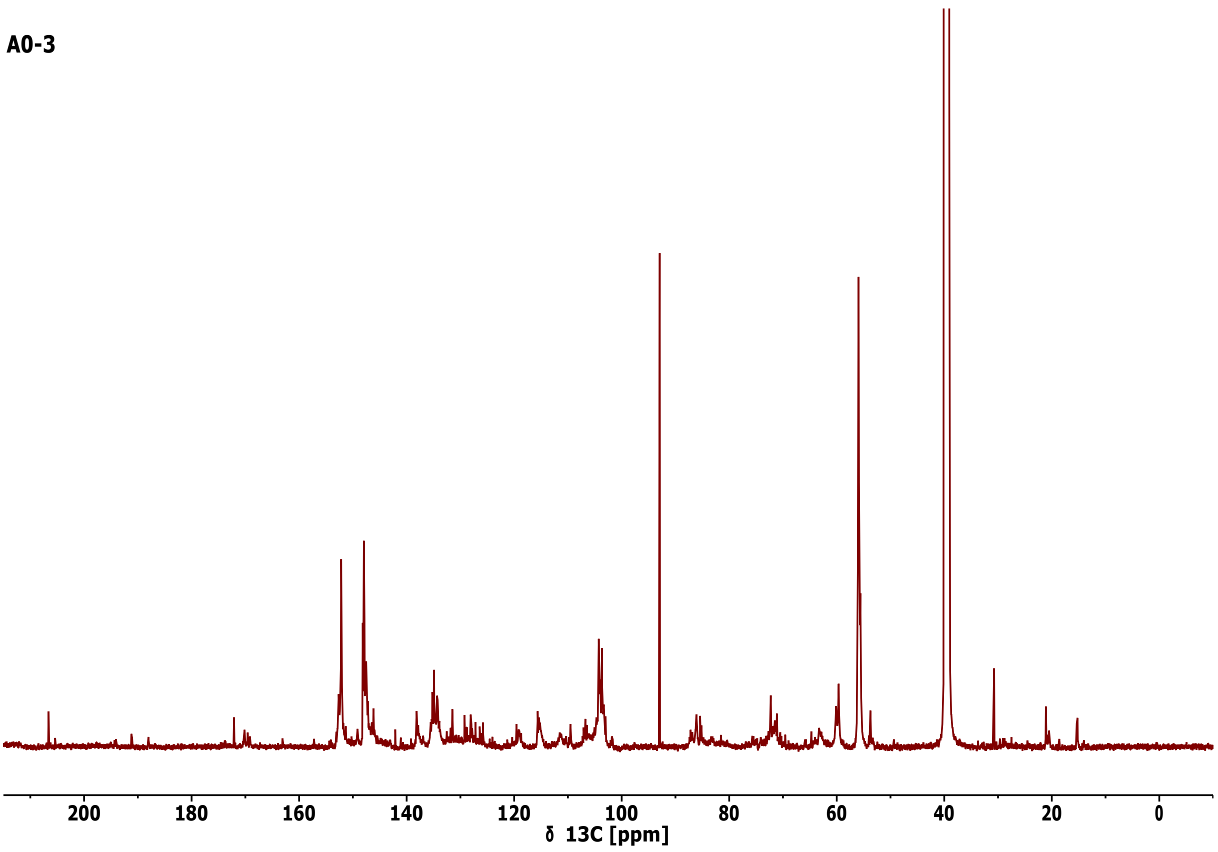

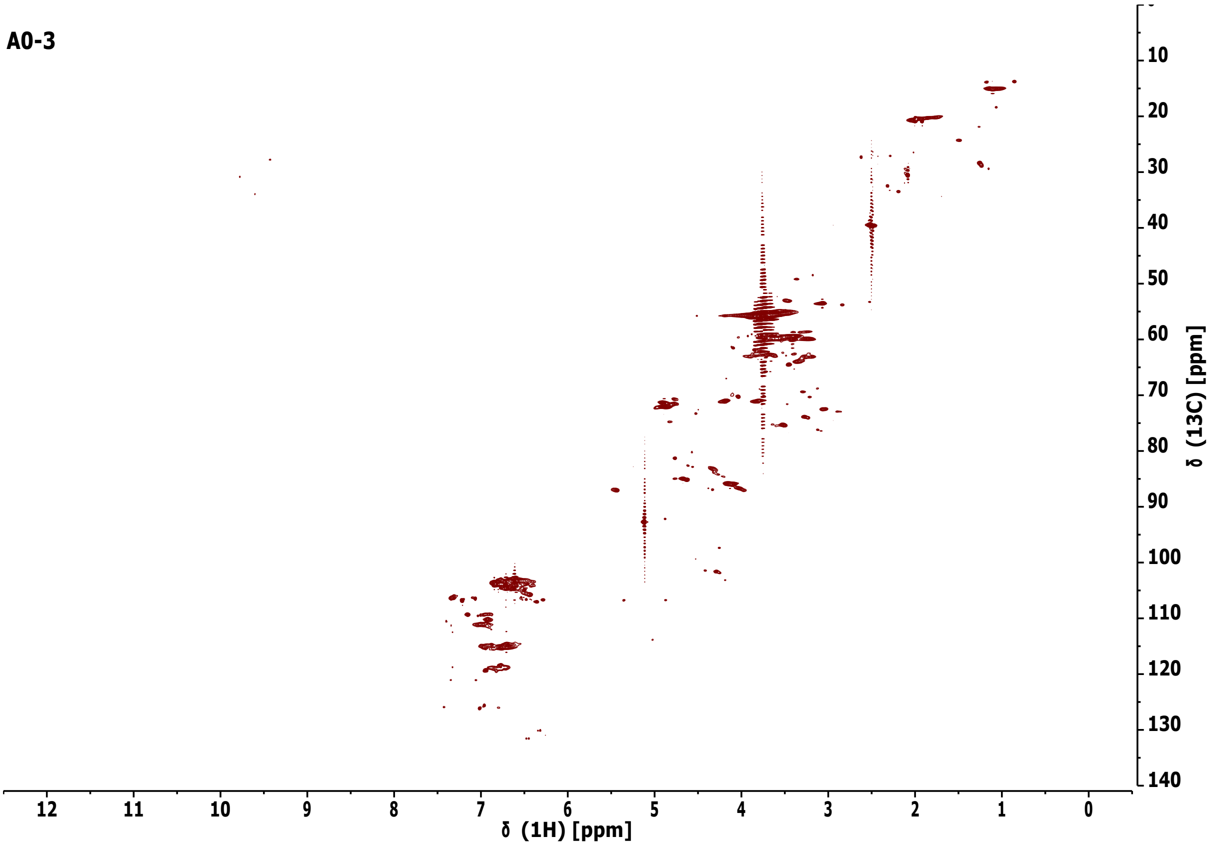

Figure S4. The ^{13}C (top) and HSQC (bottom) spectra for lignin sample **A0-3**.

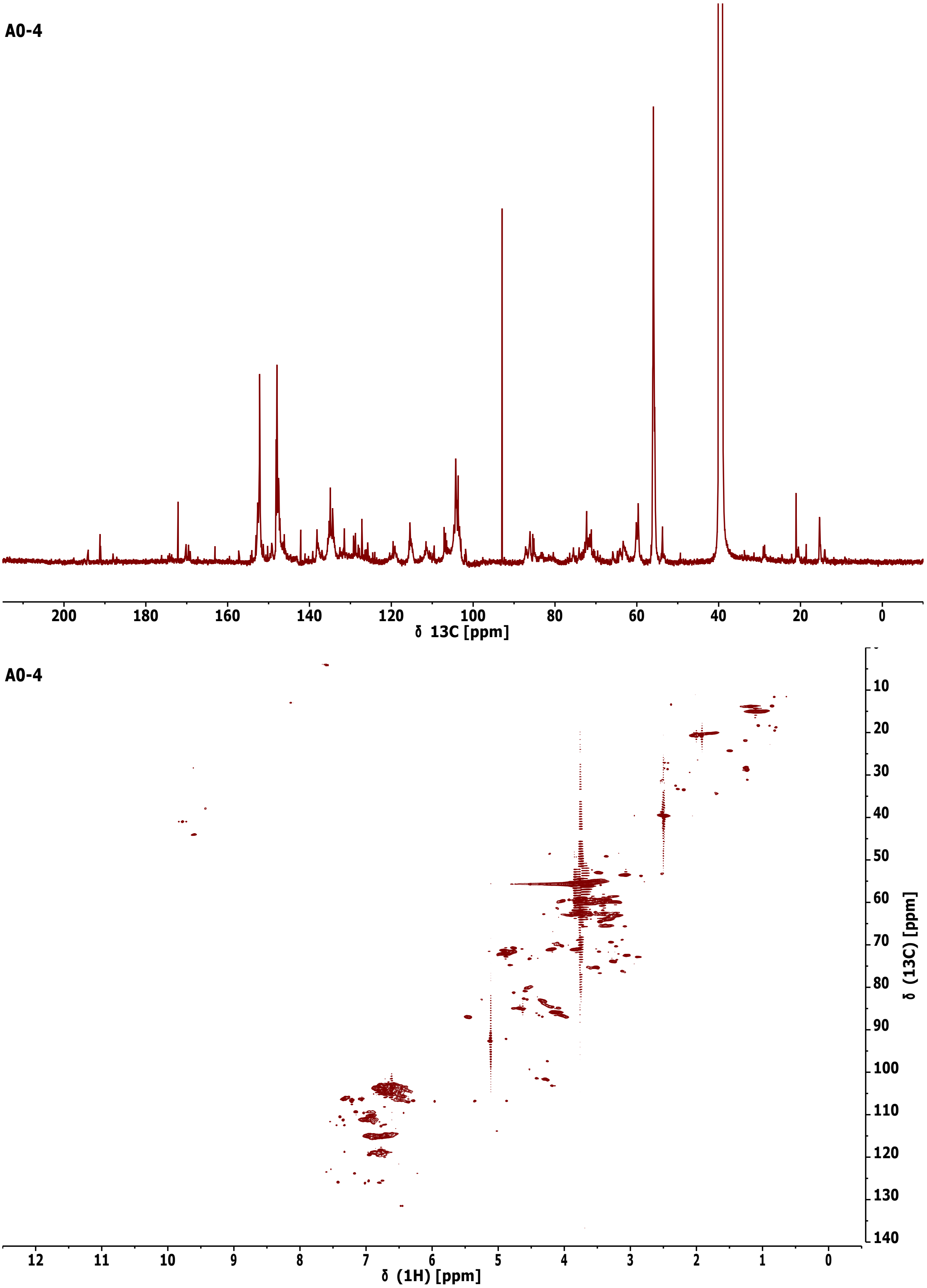

Figure S5. The ^{13}C (top) and HSQC (bottom) spectra for lignin sample **A0-4**.

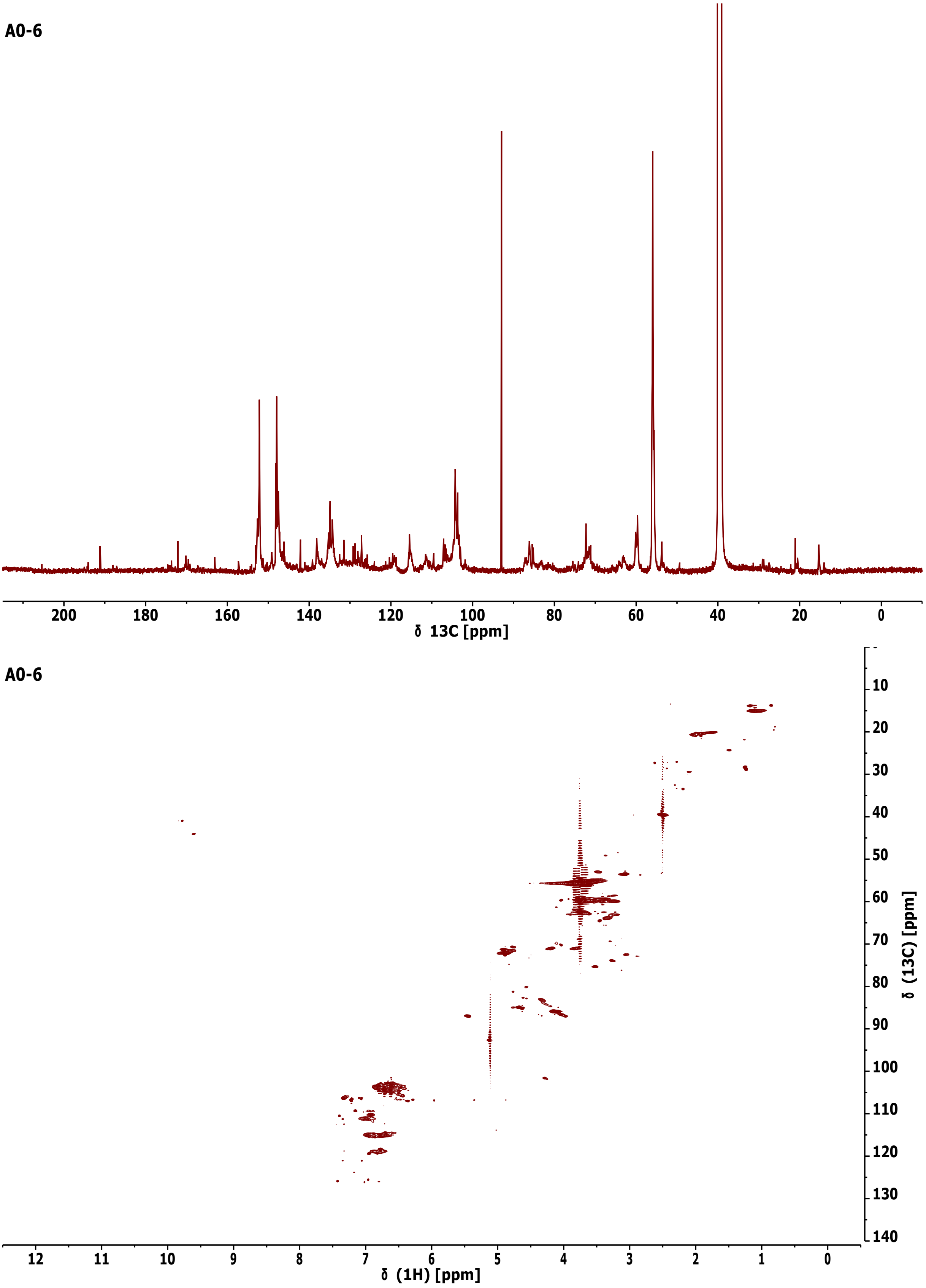

Figure S6. The ^{13}C (top) and HSQC (bottom) spectra for lignin sample **A0-6**.

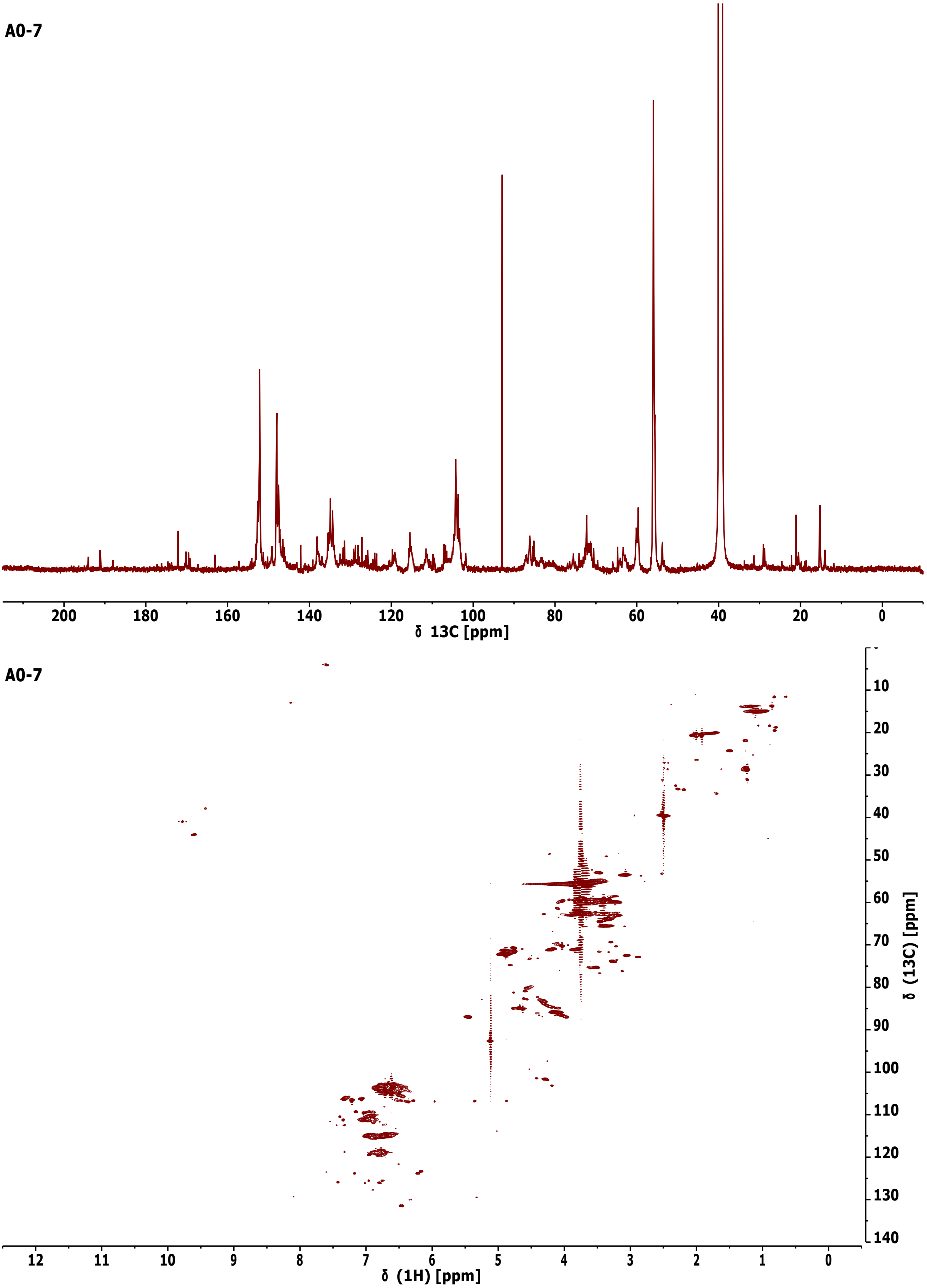

Figure S7. The ^{13}C (top) and HSQC (bottom) spectra for lignin sample **A0-7**.

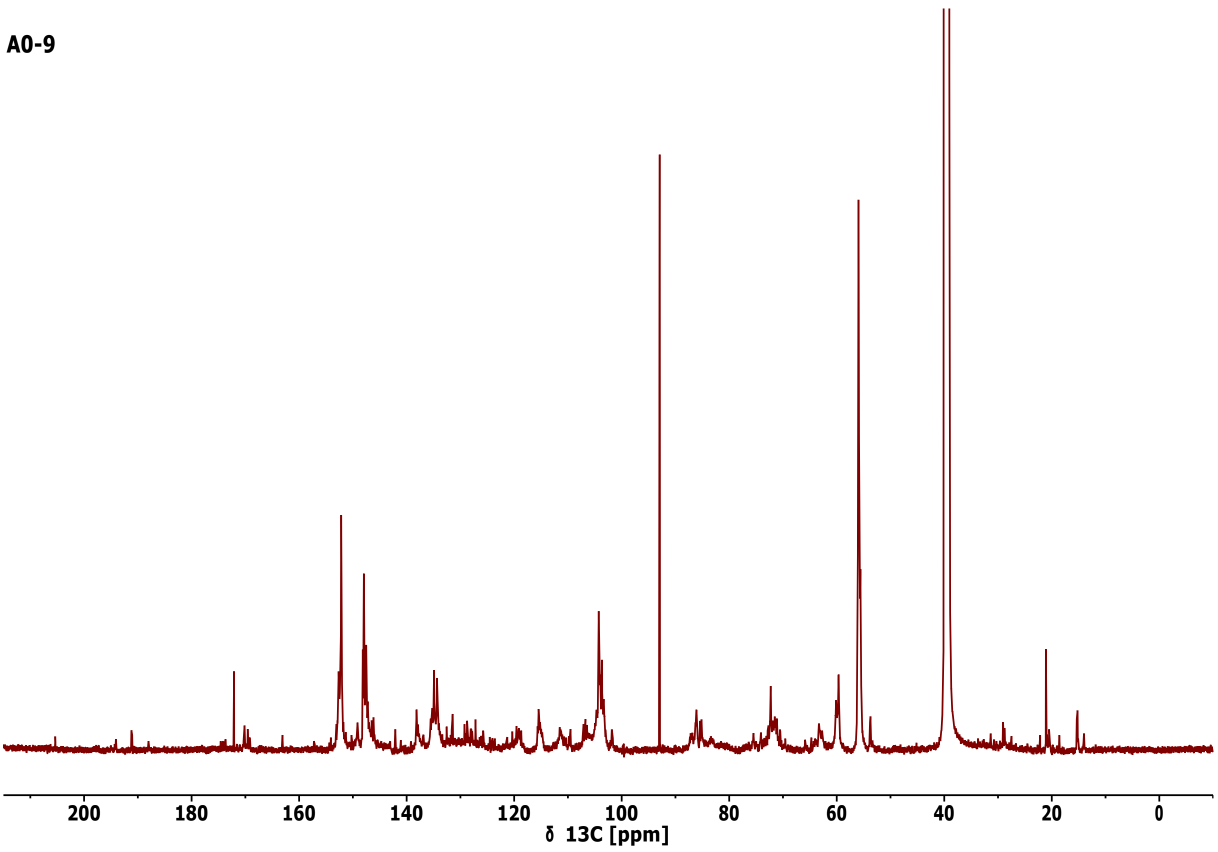

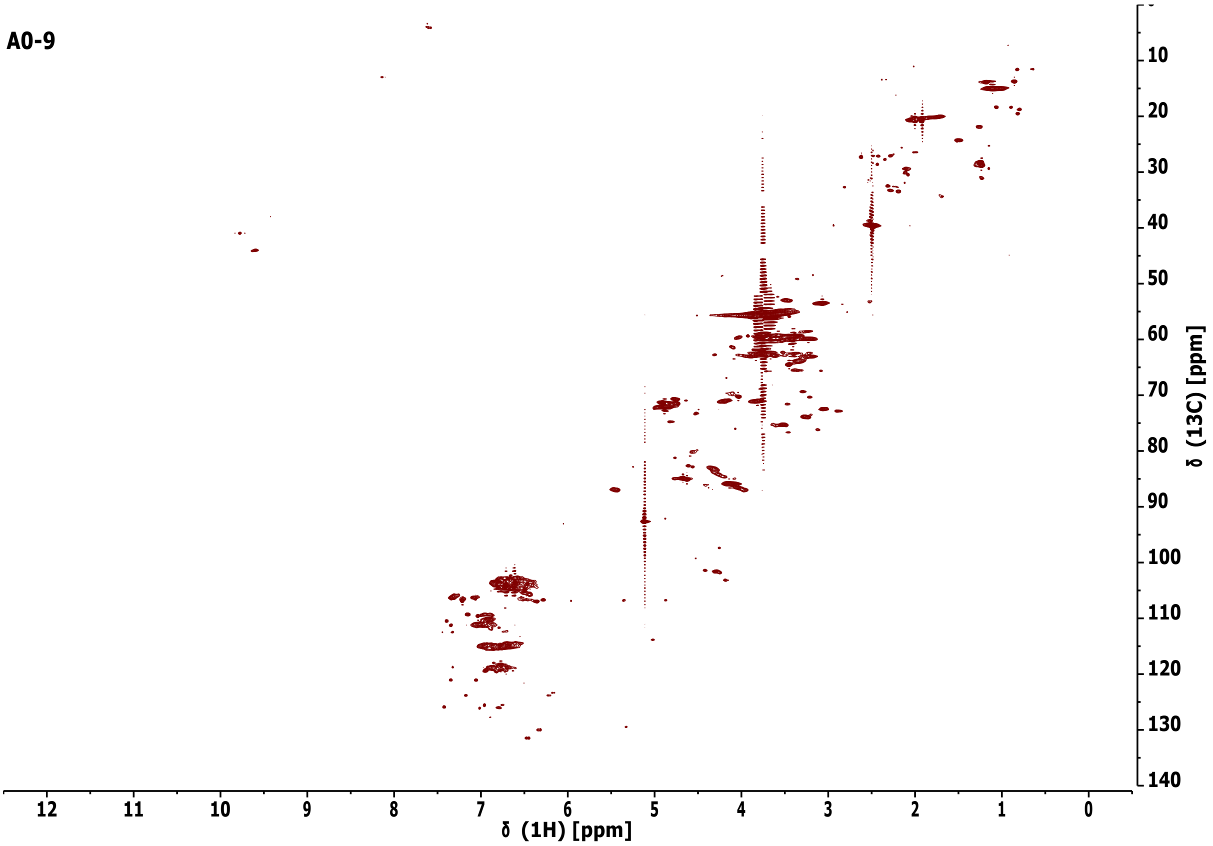

Figure S8. The ^{13}C (top) and HSQC (bottom) spectra for lignin sample **A0-9**.

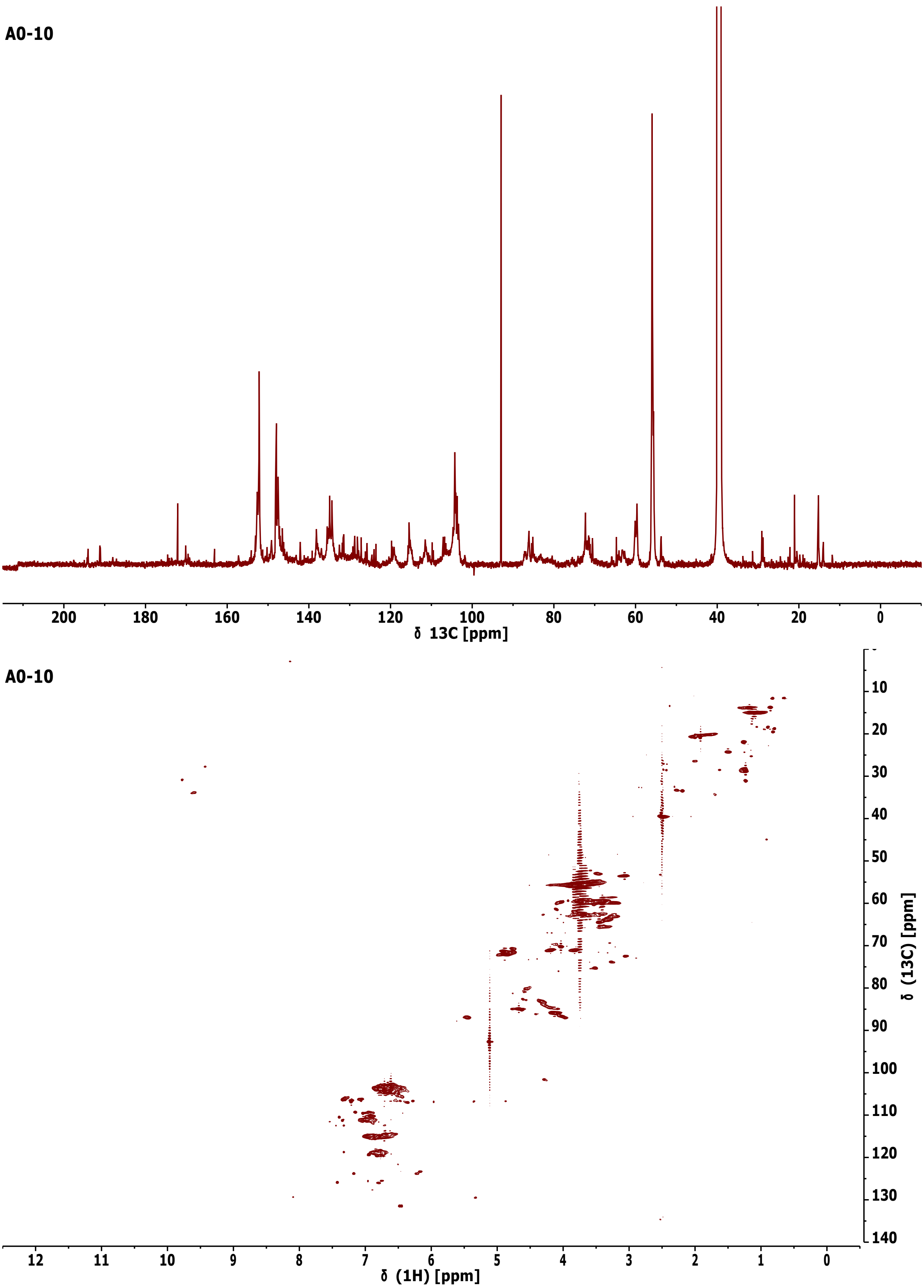

Figure S9. The ^{13}C (top) and HSQC (bottom) spectra for lignin sample **A0-10**.

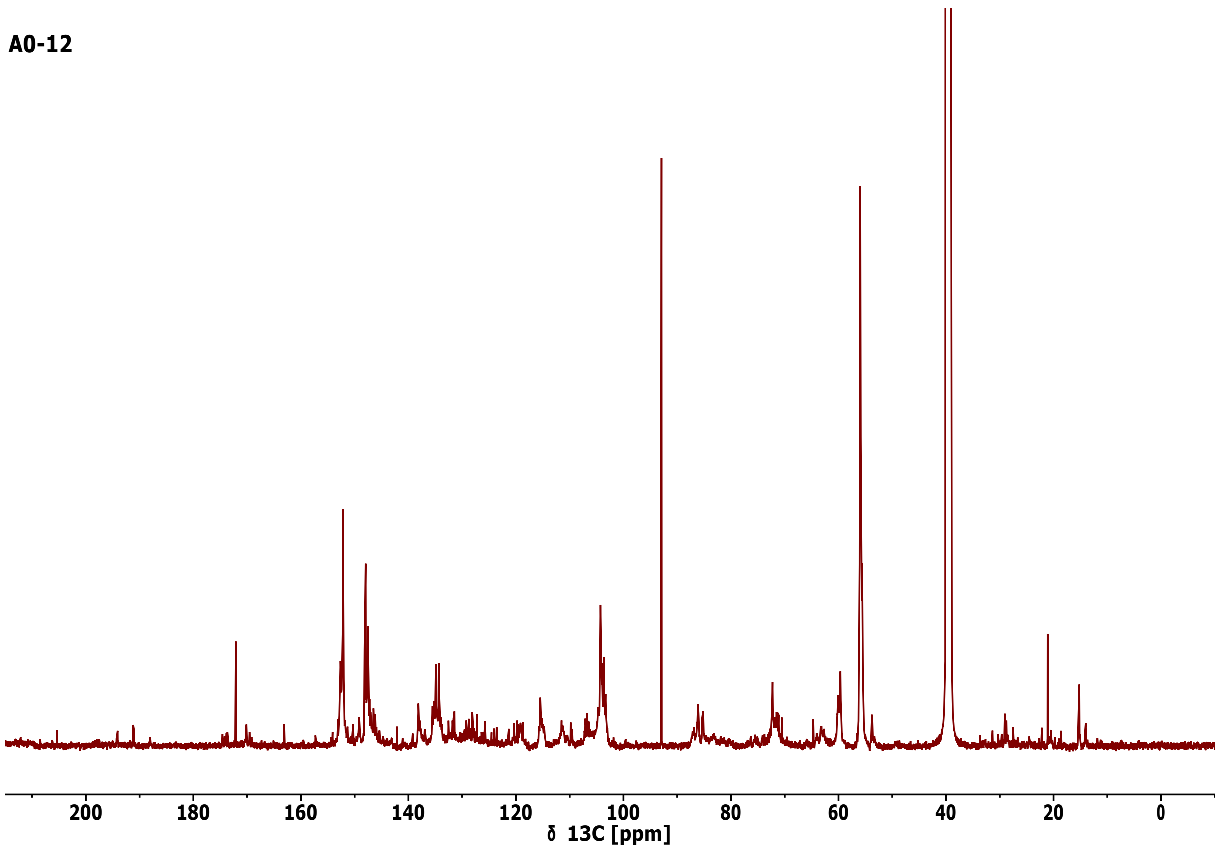

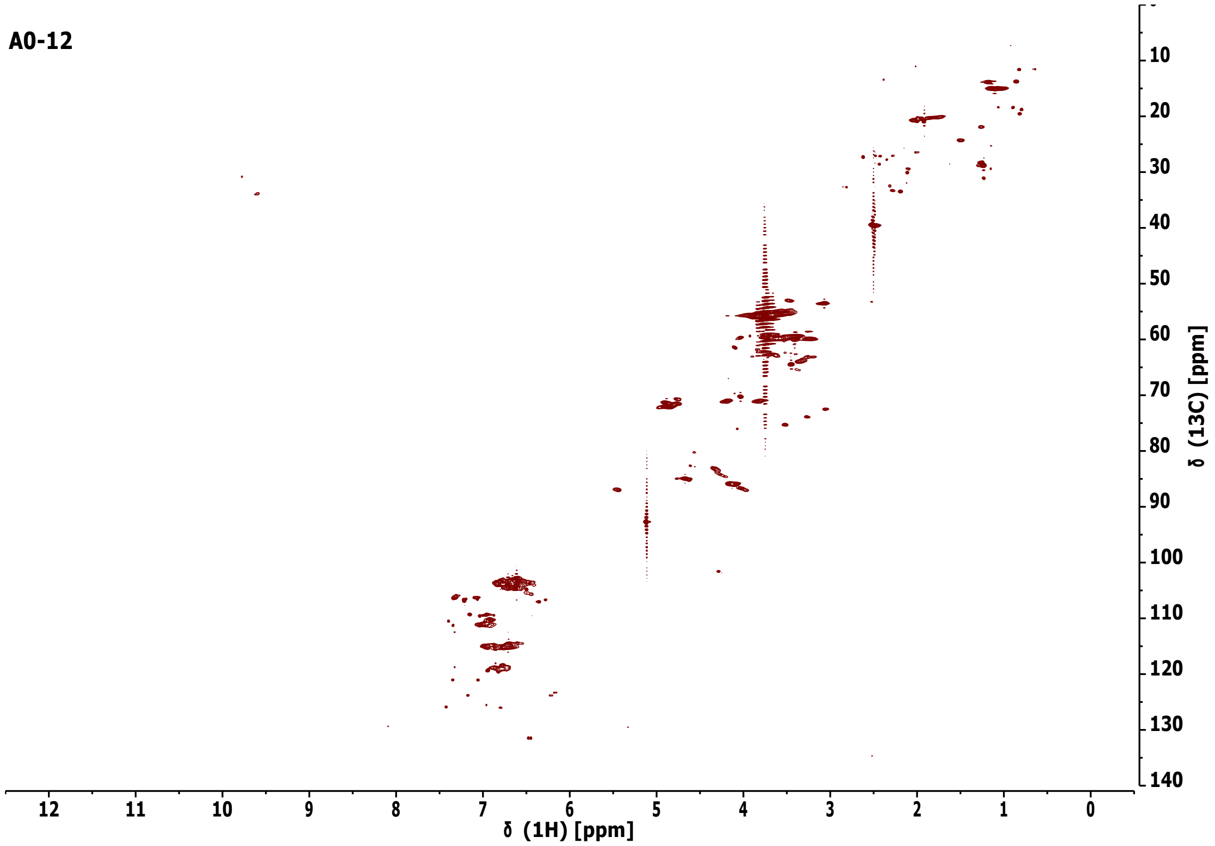

Figure S10. The ^{13}C (top) and HSQC (bottom) spectra for lignin sample **A0-12**.

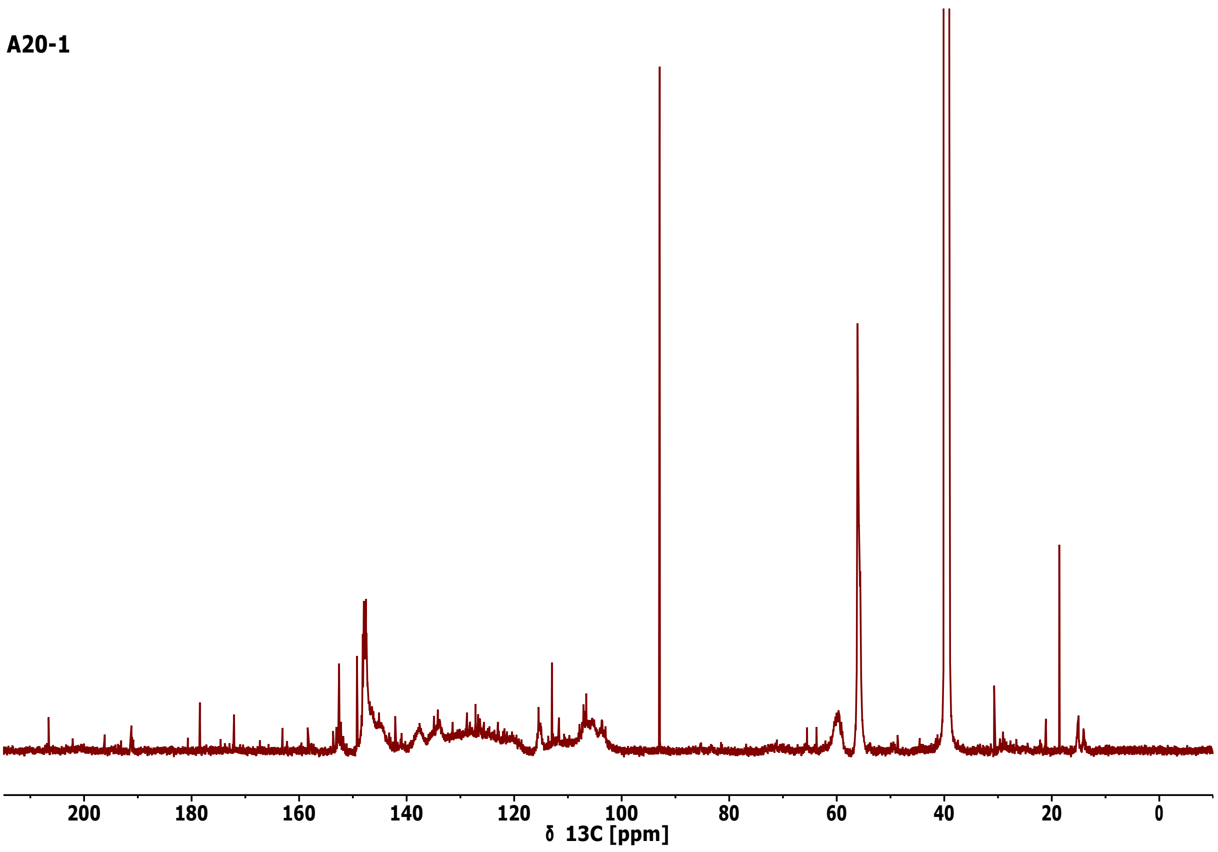

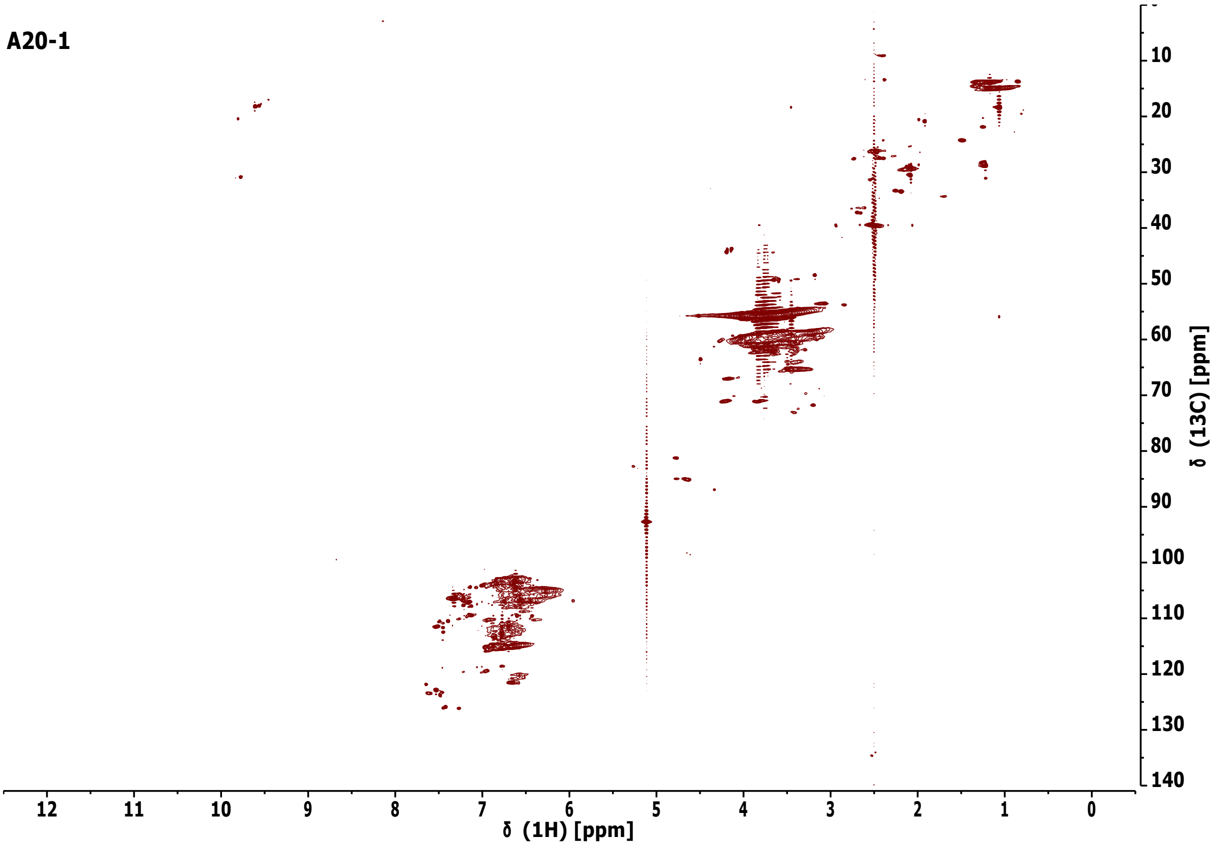

Figure S11. The ^{13}C (top) and HSQC (bottom) spectra for lignin sample **A20-1**.

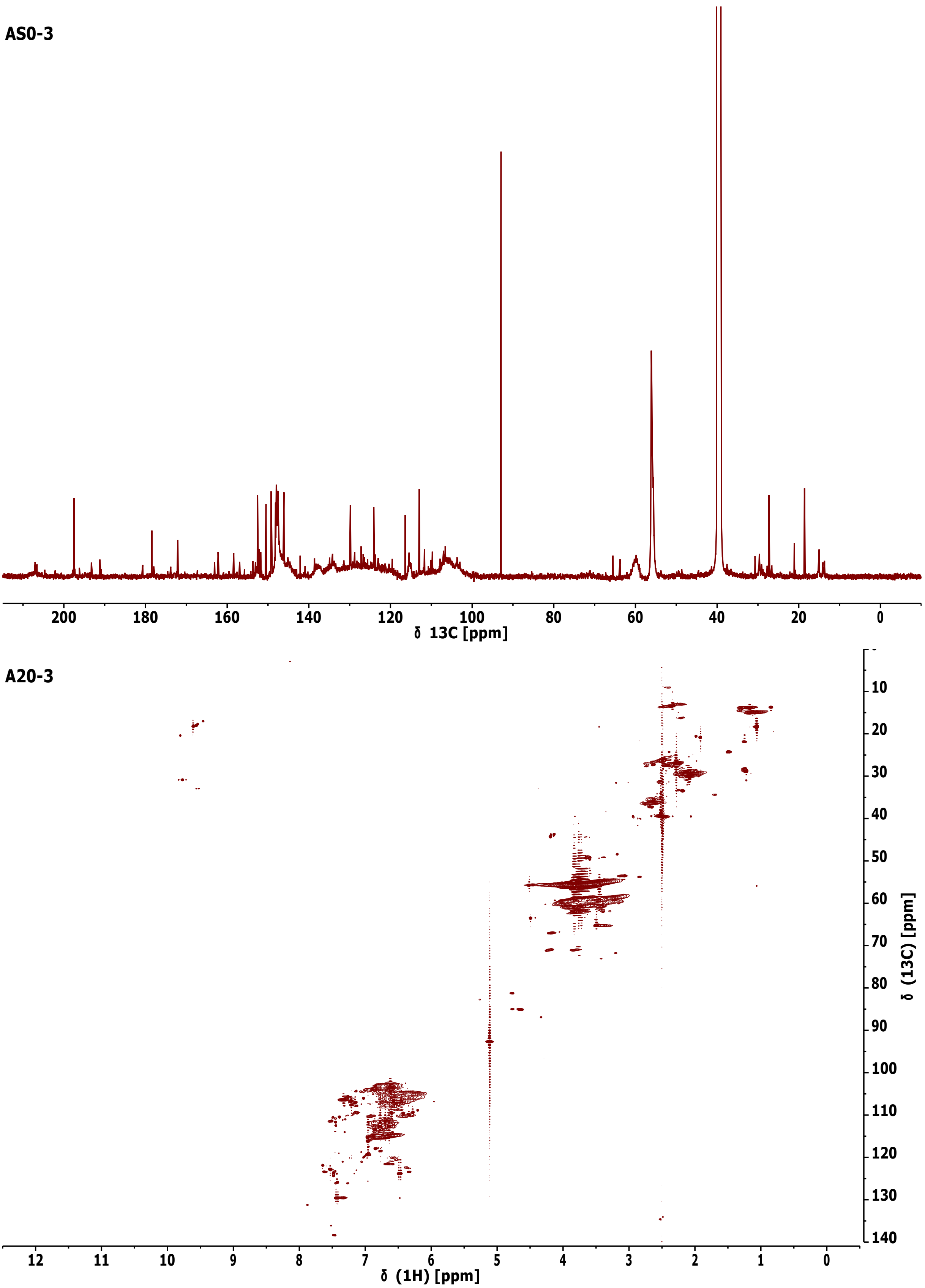

Figure S12. The ^{13}C (top) and HSQC (bottom) spectra for lignin sample **A20-3**.

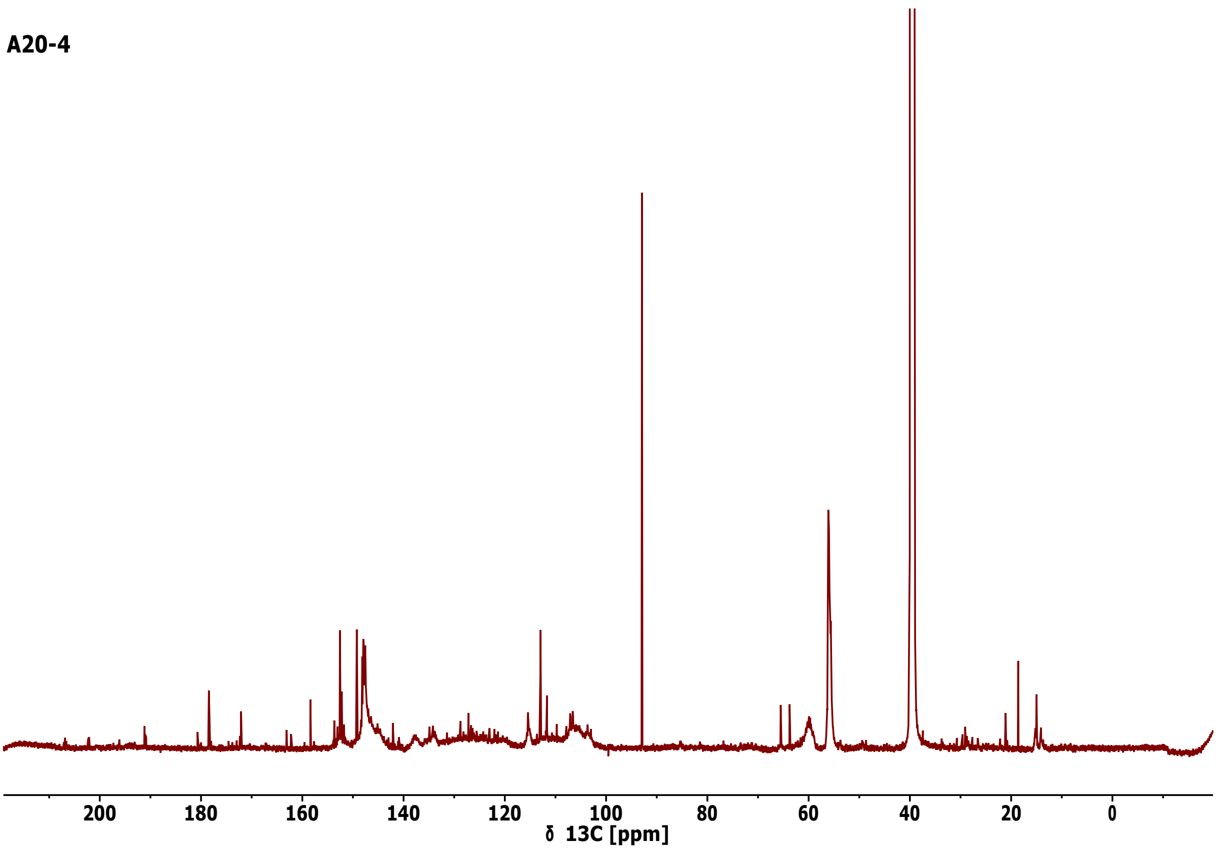

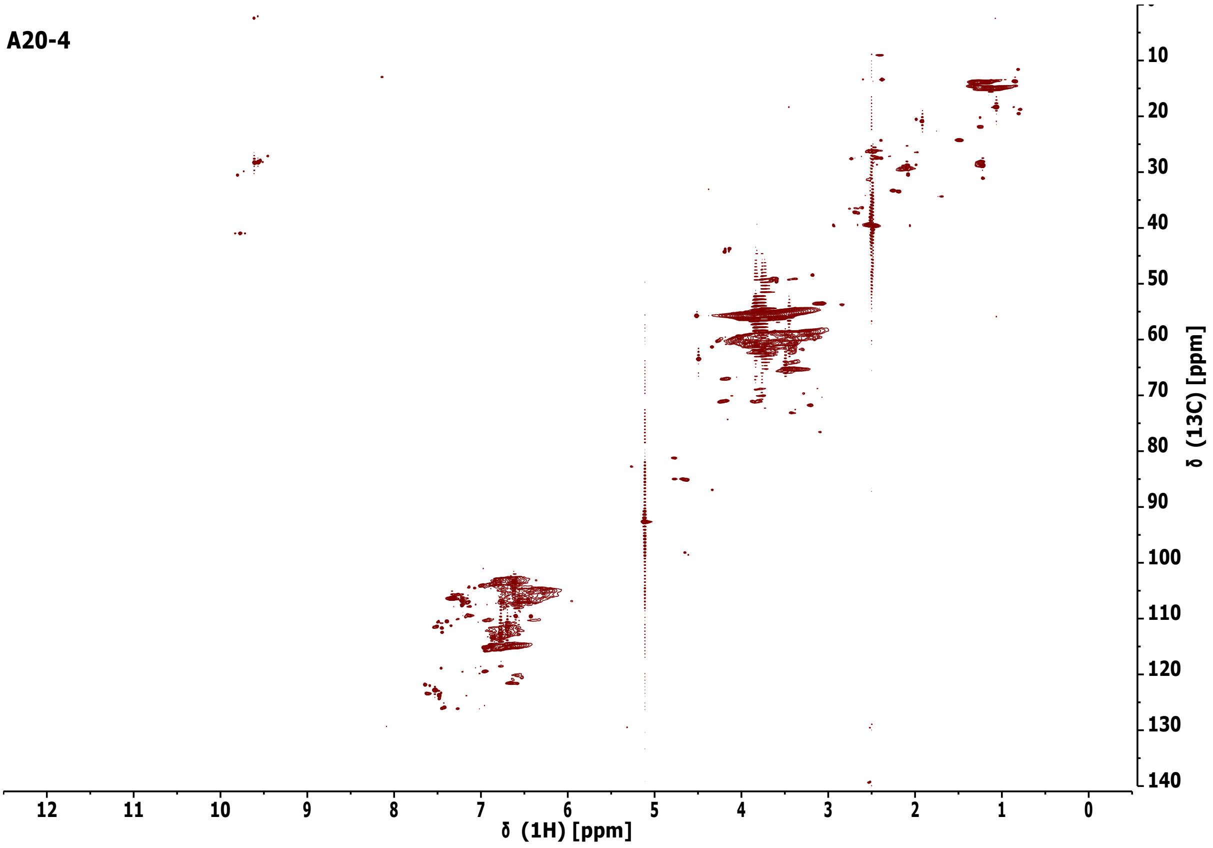

Figure S13. The ^{13}C (top) and HSQC (bottom) spectra for lignin sample **A20-4**.

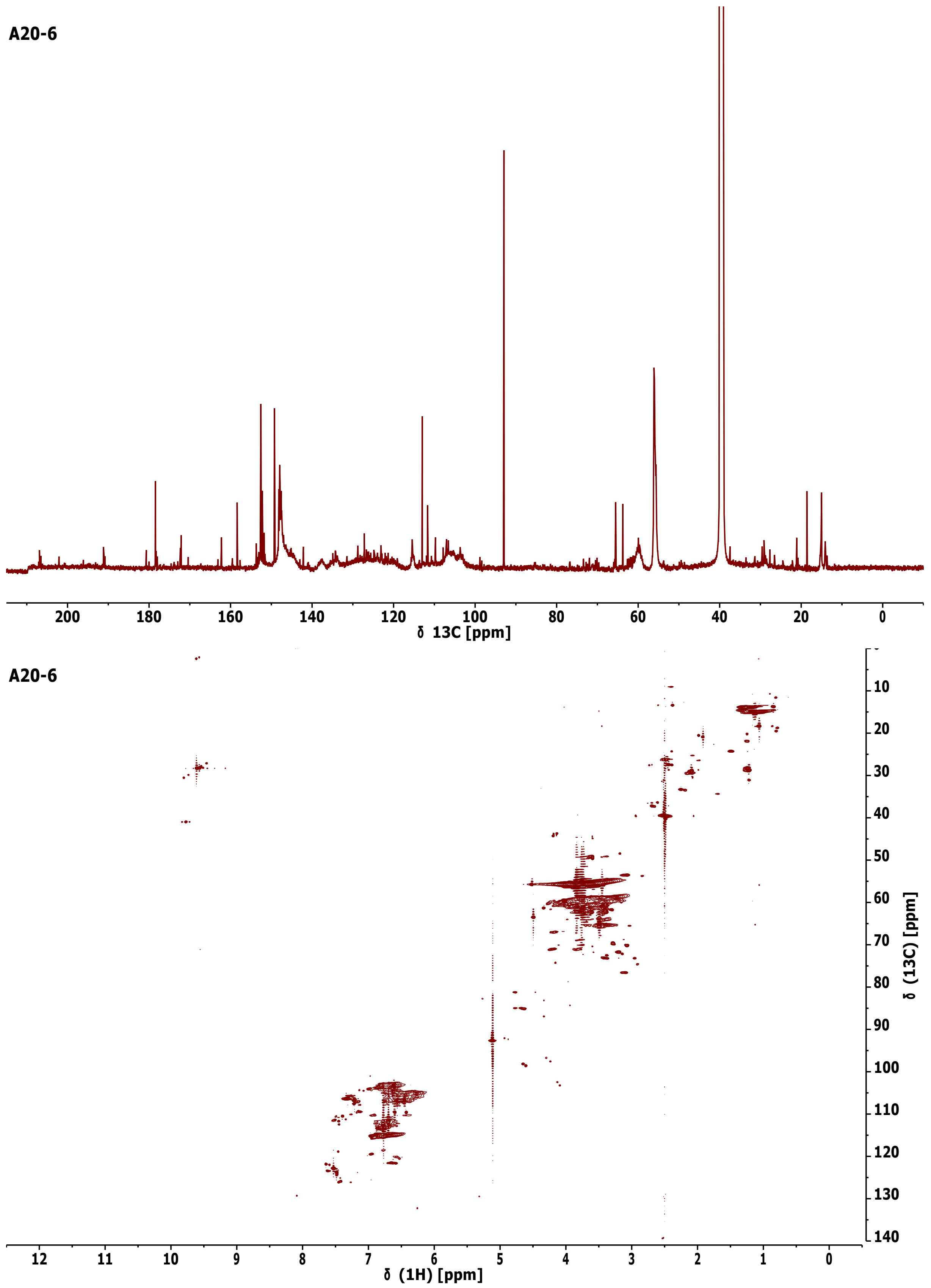

Figure S14. The ^{13}C (top) and HSQC (bottom) spectra for lignin sample **A20-6**.

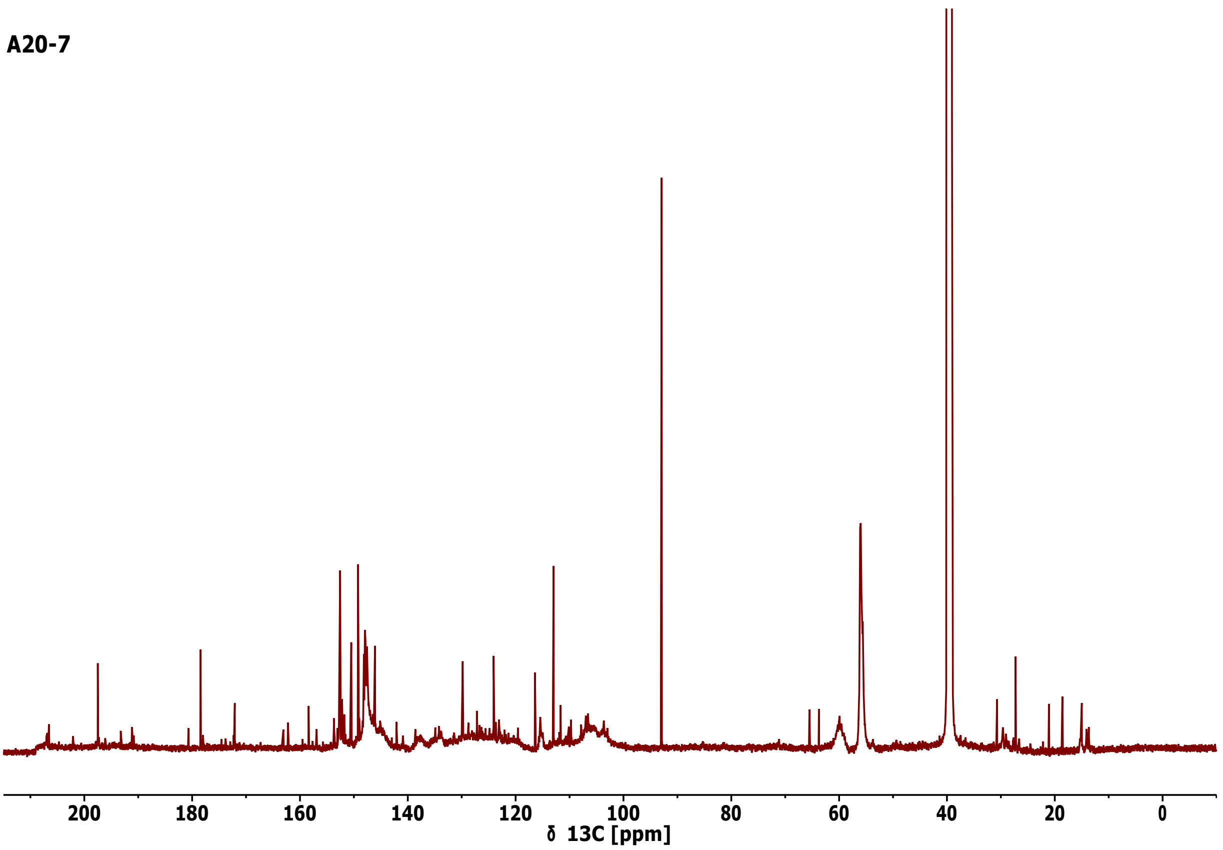

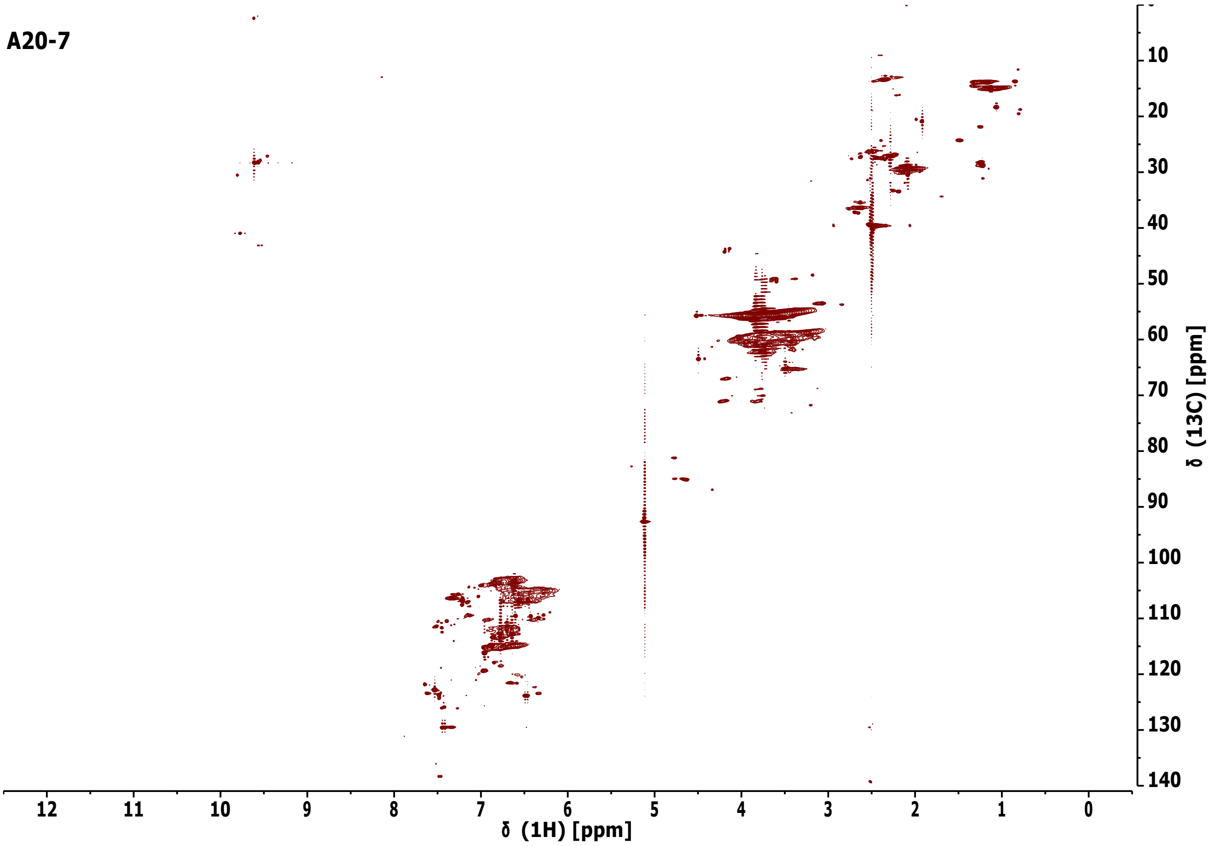

Figure S15. The ^{13}C (top) and HSQC (bottom) spectra for lignin sample **A20-7**.

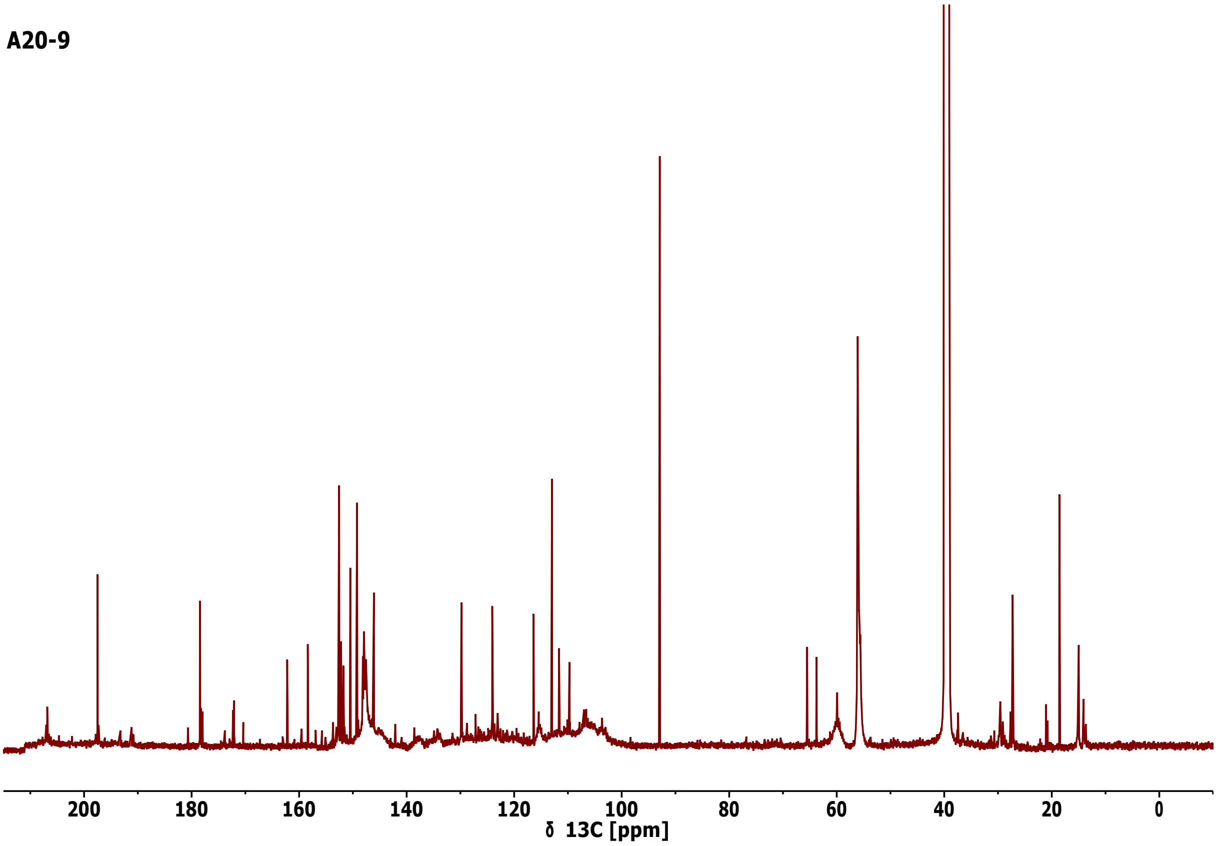

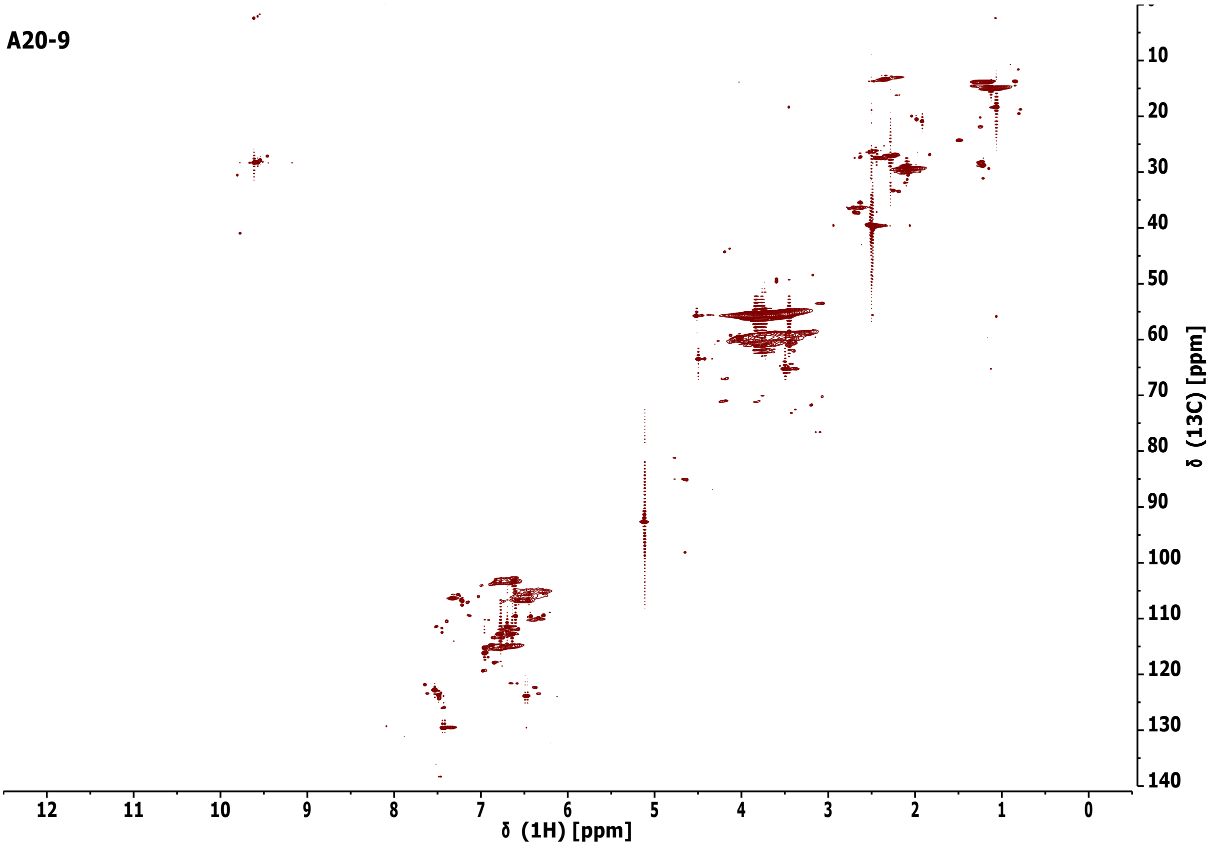

Figure S16. The ^{13}C (top) and HSQC (bottom) spectra for lignin sample **A20-9**.

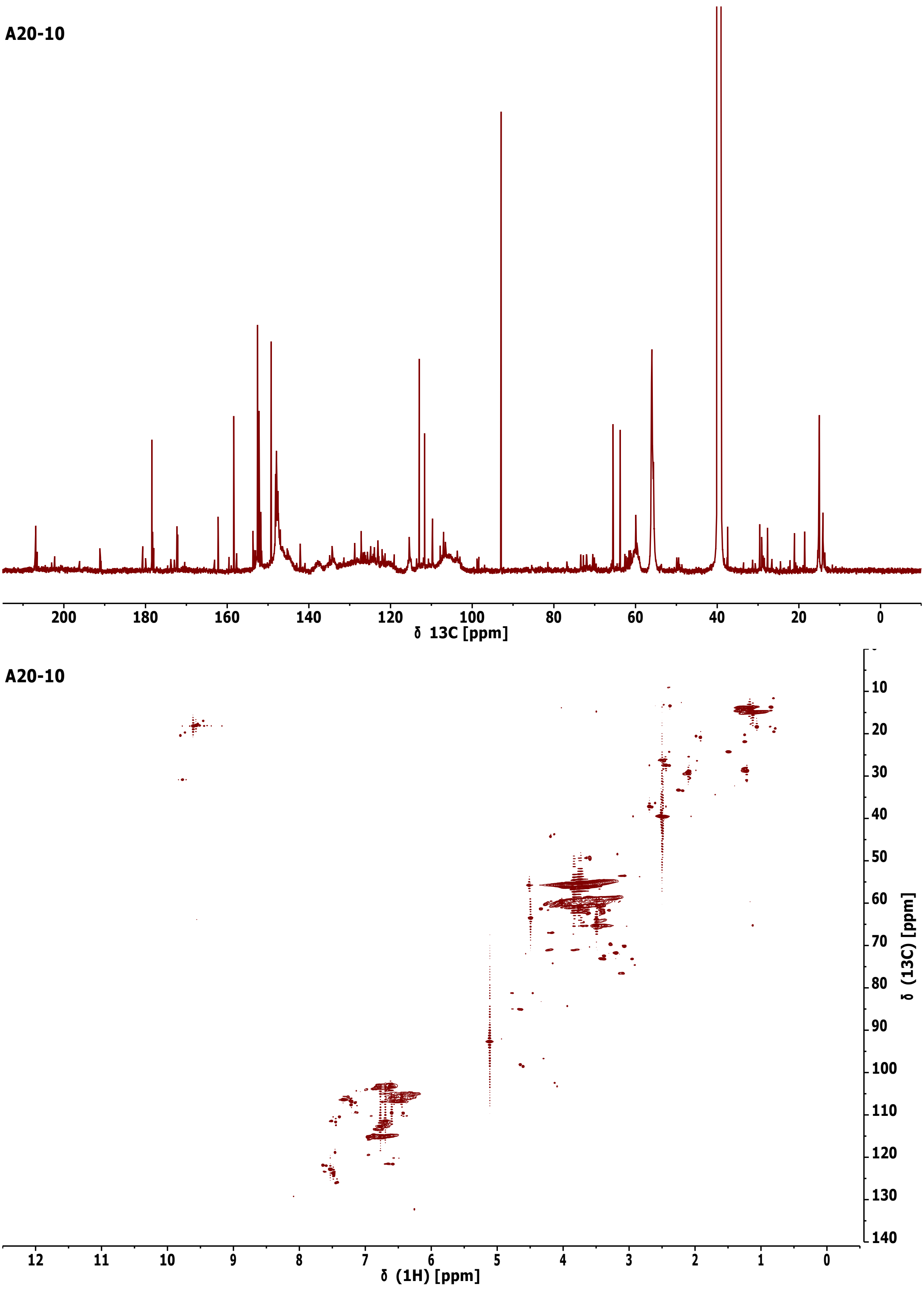

Figure S17. The ^{13}C (top) and HSQC (bottom) spectra for lignin sample **A20-10**.

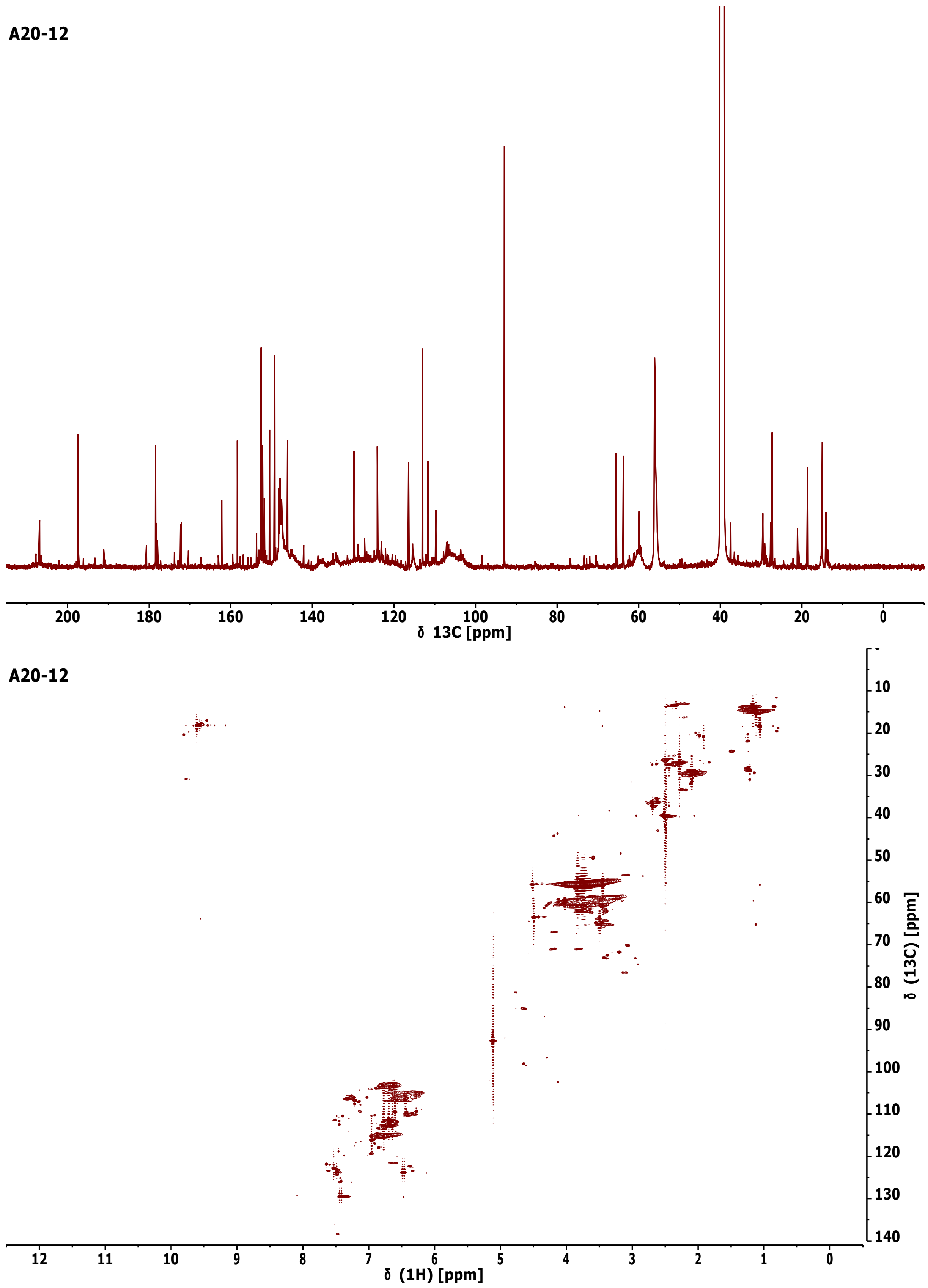

Figure S18. The ^{13}C (top) and HSQC (bottom) spectra for lignin sample **A20-12**.
